# Root rot a silent alfalfa killer in China: Distribution, fungal, and oomycete pathogens, impact of climatic factors and its management

**DOI:** 10.3389/fmicb.2022.961794

**Published:** 2022-08-11

**Authors:** Aqleem Abbas, Mustansar Mubeen, Muhammad Aamir Sohail, Manoj Kumar Solanki, Babar Hussain, Shaista Nosheen, Brijendra Kumar Kashyap, Lei Zhou, Xiangling Fang

**Affiliations:** ^1^State Key Laboratory for Managing Biotic and Chemical Threats to the Quality and Safety of Agro-products, Institute of Agro-product Safety and Nutrition, Zhejiang Academy of Agricultural Sciences, Hangzhou, China; ^2^State Key Laboratory of Grassland Agro-ecosystems, Key Laboratory of Grassland Livestock Industry Innovation, Ministry of Agriculture and Rural Affairs, College of Pastoral Agriculture Science and Technology, Lanzhou University, Lanzhou, China; ^3^Department of Plant Pathology, College of Agriculture, University of Sargodha, Sargodha, Pakistan; ^4^College of Plant Science and Technology, Huazhong Agricultural University, Wuhan, China; ^5^Faculty of Natural Sciences, Plant Cytogenetics and Molecular Biology Group, Institute of Biology, Biotechnology and Environmental Protection, University of Silesia in Katowice, Katowice, Poland; ^6^Department of Plant Sciences, Karakoram International University, Gilgit, Gilgit Baltistan, Pakistan; ^7^Colin Ratledge Center for Microbial Lipids, School of Agriculture Engineering and Food Science, Shandong University of Technology, Zibo, China; ^8^Department of Biotechnology Engineering, Institute of Engineering and Technology, Bundelkhand University, Jhansi, India

**Keywords:** alfalfa, root rot, China, distribution, climatic factors, management

## Abstract

Alfalfa plays a significant role in the pasture ecosystems of China’s north, northeast, and northwest regions. It is an excellent forage for livestock, improves soil structure, prevents soil erosion, and has ecological benefits. Presently root rot is a significant threat to the alfalfa productivity because of the survival of the pathogens as soil-borne and because of lack of microbial competition in the impoverished nutrient-deficient soils and resistant cultivars. Furthermore, these regions’ extreme ecological and environmental conditions predispose alfalfa to root rot. Moisture and temperature, in particular, have a considerable impact on the severity of root rot. Pathogens such as *Fusarium* spp. and *Rhizoctonia solani* are predominant, frequently isolated, and of major concern. These pathogens work together as disease complexes, so finding a host genotype resistant to disease complexes is challenging. Approaches to root rot control in these regions include mostly fungicides treatments and cultural practices and very few reports on the usage of biological control agents. As seed treatment, fungicides such as carbendazim are frequently used to combat root rot; however, resistance to fungicides has arisen. However, breeding and transgenic approaches could be more efficient and sustainable long-term control strategies, especially if resistance to disease complexes may be identified. Yet, research in China is mainly limited to field investigation of root rot and disease resistance evaluation. In this review, we describe climatic conditions of pastoral regions and the role of alfalfa therein and challenges of root rot, the distribution of root rot in the world and China, and the impact of root rot pathogens on alfalfa in particular *R. solani* and *Fusarium* spp., effects of environmental factors on root rot and summarize to date disease management approach.

## Introduction

Alfalfa (*Medicago sativa* L.,) is a perennial legume and has long been utilized as a forage crop due to its high yield, nutritional value and adaptability to wide range of soils and under various climatic conditions (Khoury et al., 2014; Luo et al., 2014; Jiang et al., 2021). In China, alfalfa is mainly grown for feeding grazing livestock (Jiang et al., 2021). Root rot is the major limiting factor to alfalfa production worldwide. It is estimated that the annual yield loss worldwide caused by root rot is 20 to 40% (Wang et al., 2020a). Root rot is a destructive disease complex caused by several plant pathogenic fungi and oomycetes. Among these pathogens, *Fusarium* spp., and *Rhizoctonia solani* are the most damaging and frequently occurring root rot pathogens (Anjanappa et al., 2016; Cao et al., 2020; Cai et al., 2021; Jiang et al., 2021; Xu et al., 2021; Zhao et al., 2021). In China, root rot is endemic in major alfalfa producing regions such as northwest China (Gansu, Xinjiang, Qinghai, Shaanxi, and Ningxia Huizu), north China (Hebei, Shanxi, and Inner Mongolia), and northeast China (Heilongjiang, Liaoning, and Jilin), with death rates of more than 60% on severe plots (Fang et al., 2019, 2021). The root-specific symptoms go unreported or are not visible, and plants that show symptoms aboveground do not recover. Root lesions of various sizes and colors (reddish, brownish, and blackish) and browning and weakening of root tips, yellowing and wilting of leaves, slowed plant growth, lower yield, and crop loss are some of the symptoms associated with root rot (Fang et al., 2019). Environmental factors in particular soil temperature and soil moisture have profound effects on the expression and severity of root rot and the consequent productivity of alfalfa (Yinghua et al., 2019; Sharath et al., 2021). In response to root rot, researchers in China have carried out a series of research, focusing mainly on the investigation of the occurrence and severity of the disease, the identification and isolation of the pathogen, the determination of pathogenicity and biological characteristics, evaluation of alfalfa species resistance and cultural, chemical, and biological control (Fang et al., 2021; Zhang et al., 2021). These attempts, however, have only been somewhat successful (Pan et al., 2015; Zhang et al., 2021). Furthermore, it is hard to develop a resistant cultivar resistant to diverse pathogens. Subsequently, farmers rely only on chemical control; nonetheless, fungicide resistance has been recorded. Due to a lack of understanding about the disease and the dire threat root rot poses to the alfalfa in China, there is a pressing need to research the biology, ecology, epidemiology, and management of root rot. This review: (i) describes the climatic conditions of pastoral regions of China and role alfalfa therein and challenges from root rot; (ii) discusses the distribution of root rot in the world and China; (iii) addresses the status of root rot in the world and China mainly focusing *Fusarium* spp. and *R. solani*, describes the disease cycle and biological characteristics including the symptoms they caused; and the challenges posed to alfalfa from these pathogens; (iv) addresses the environmental factors affecting the severity of the root rot; (v) addresses the approaches on the disease management made to date using cultural, breeding and transgenic, biological, chemical, gene silencing and editing.

## Climatic condition of pastoral regions of China, role of alfalfa there in and the challenges from root rot

The climate of the northwest, north, and northeast region of China, where alfalfa is grown can best be described as “an arid, semi-arid and subhumid climate characterized by plenty of water but low solar radiation in the northeast, with a temperate climate, low precipitation and scarce water in north China and vast areas of low-quality land, abundant solar radiation and thermal resources, scarce water, desertification and salinization in the northwest (Morrison, 2004; Liang et al., 2006). In some locations of the northwest, water is plentiful but unevenly distributed (Su et al., 2004). The soil of these regions has originated from nutrient-poor, ancient parent materials that have been intensively weathered and leached (Delang, 2018). Besides, the soil is not very fertile and generally lacking in phosphorus, resulting in significantly reduced nutrient levels in particular northwest and north region (Wang et al., 2021). Alfalfa is the most famous perennial leguminous forage in the world. The United States is the world’s largest alfalfa producer with a planting area of 9 million hectares, followed by Argentina with a planting area of 6.9 million hectares. China ranks fifth globally with a planting area of 4.7 million hectares (Bao et al., 2016; Gao et al., 2018a; Guo et al., 2019). In China, alfalfa is the most crucial pasture legume in the northwest, northeast, and north regions. In the northwest, alfalfa is sown over an estimated area of 3151.9 thousand hm^2^, accounting for 66.43% of the country’s total area. North China is the second-largest alfalfa producing area in China, and alfalfa is sown over an estimated area of 1063.4 thousand hm^2^, accounting for 22.41% of China’s total area. Northeast alfalfa acreage accounted for a small proportion in China, with a planting area of only 234.5 thousand hm^2^, accounting for 4.94% of China (Feng and Kai, 2014 Shi et al., 2017). In recent years, the Chinese government has gradually allowed more land for forage crops such as alfalfa. Hence, alfalfa has become the most widely used forage legume in China’s integrated farming systems, grazing, and ecological conservation. Besides, the concept of “pasture-based livestock industries” alfalfa has gained attention in recent years, its planting area ranks first among all other forage crops reaching over almost 11 million acres by the end of 2017 (Shi et al., 2017). Compared to other forages and grain legumes like soybeans, alfalfa is the “queen of forage,” providing dairy with digestible protein, fiber, minerals, and vitamins at meager costs. While dairy is a relatively young agricultural business in China, with much lower average yields than in many other countries, boosting alfalfa consumption has been identified as a critical approach for improving milk supply and quality, particularly for big dairy farms. Large dairy farms in China have increased significantly (Qingbin and Yang, 2020). The advantages of alfalfa as a perennial pasture include its outstanding ability to prevent soil and wind erosion. In addition, extensive and deep root system of alfalfa is highly effective for building up organic matter, improving soil structure and soil fertility (Liu et al., 2013; Jun et al., 2014). Additionally, alfalfa is relatively tolerant to water deficit and water loggings. Besides, alfalfa is widely used for livestock and poultry, water and soil conservation, green manure, and disease breaks. However, continuous cropping of alfalfa over time in these regions has caused severe soil water deficit, soil desiccation, and depletion of shallow groundwater (Wang et al., 2021). Besides frequent droughts, soil moisture deficit, erratic precipitation, severe wind, and water erosions, and intensive grazing have made alfalfa vulnerable to root rot. Furthermore, the poor and nutrient-deficient soils, in particular phosphorus, these regions predispose alfalfa plants to root rot because microbial competition with the root rot pathogen in such conditions is generally lacking and, consequently, losses from root rot are exacerbated (Wang et al., 2016; Feng et al., 2020). Besides, cool soil temperature and soil compaction in these regions slows down alfalfa growth and predispose roots to root rot. Alfalfa can survive for ten years or more years; however, once infected by root rot, yield reduction starts even in the third or second year (Fang et al., 2019). Besides, toxins such as mycotoxins and phytotoxins produced by root rot pathogens may also pose a substantial danger to feed quality due to their effects on animal productivity and potentially on human food quality. In addition, increased costs and harmful effects of fungicides are the indirect losses due to root rot (Barbetti et al., 2007). Furthermore, root rot pathogens form pathogen complexes, thereby posing a synergistic influence on the severity of root rot (Gossen et al., 2016; Fang et al., 2019). These pathogen complexes respond differently to fungicides and other management practices, and the root rot they cause requires special treatment measures.

## Distribution of root rot in the world and China

Root rot has seriously affected alfalfa production in the United States, Italy, China, Canada, Australia, Russia, Japan, and Argentina (Akamatsu et al., 2008; Yinghua et al., 2019). In Alberta and British Columbia, root rot of alfalfa was first recognized by McKenzie and Davidson (1975). About 60% production area was affected by root rot (McKenzie and Davidson, 1975). The primary pathogens responsible were *R. solani*, *Phoma sclerotioides*, *F. roseum,* and *Phytophthora megasperma*. In 1983, the incidence of crown and root rot of alfalfa was recorded in 24 alfalfa-growing areas in southern Alberta, Canada, and the average incidence was 61%, and the highest was 80%. Most of the alfalfa plants were either dead or withered, resulting in reduction of the yield and quality. The main pathogens were *F. solani*, *F. tricinctum*, *F. avenaceum*, *F. oxysporum* and *Pythium irregular.* In 1984–1987 a survey of alfalfa fields in the northeast and northwest Alberta, Canada, revealed that *F. roseum* and *F. avenaceum* were common pathogens associated with root rot (Hwang and Flores, 1987; Hwang et al., 1989). In contrast, research in Quebec, Canada, in the late 1980s revealed alfalfa fields were severely affected by *Phytophthora* spp., particularly in low-lying places, since the humid climatic condition was more conducive to proliferation and multiplication of *Phytophthora* spp. (Richard and Martin, 1991). In 1991, root rot affected alfalfa production in Nevada, United States, and the primary pathogens were *Fusarium* spp. (Snyder et al., 1991). Later in Wyoming and then in Idaho, United States, *Phoma sclerotioides* were first identified as the cause of widespread winterkill of alfalfa (Gray et al., 1997; Al-Sadi and Deadman, 2010; Al-Sadi, 2021). Subsequently, the root rot was successively found in all alfalfa-producing areas of the United States (Hollingsworth et al., 2003; Larsen et al., 2007). In 2006, *Fusarium semitectum* was identified as a major pathogen responsible for causing root rot in Italy (Garrett, 1970; Zaccardelli et al., 2006). Besides many reports on root rot from the United States, Canada, and Italy, root rot has also been reported in New Zealand, Japan, Russia, Australia, India, Brazil, Egypt, Nigeria, Finland, and many other countries (Snyder et al., 1991; Sharath et al., 2021). Hence, numerous root rot pathogens have been shown to have varying degrees of direct involvement in causing root rot disease of alfalfa in the world. Likewise, the distribution of root rot pathogens associated with root rots is influenced by environmental factors such as moisture and temperature. For example, *F. pseudograminearum* was more widespread during the low-rainfall years in the Pennsylvania of the USA and the low rainfall regions of Australia. Whereas *F. culmorum* was predominant in the high rainfall areas of eastern Australia and in the cooler and higher altitude areas of Idaho, USA (Smiley and Yan, 2009).

**Figure 1:**
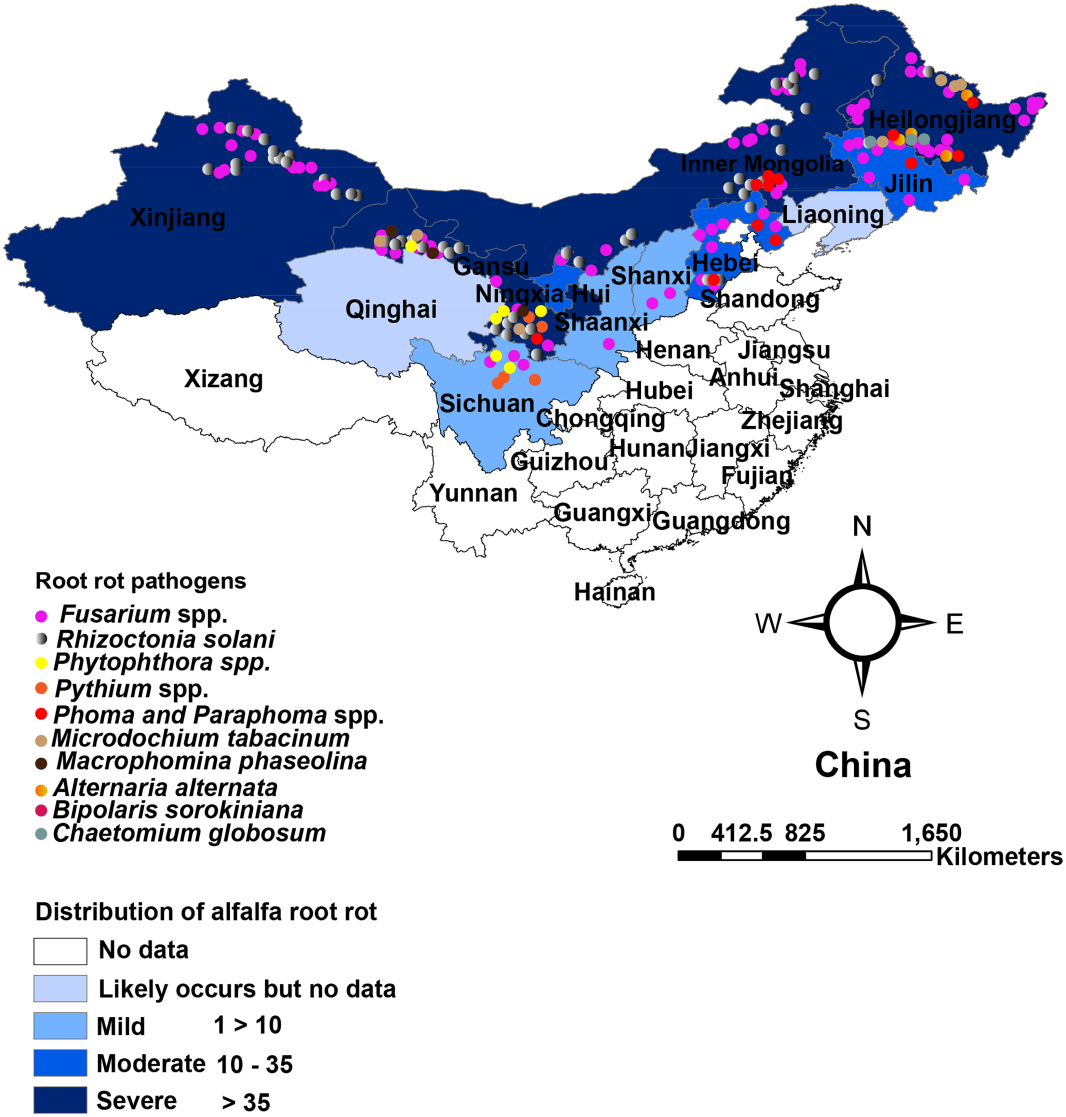
Geographical distribution and affected region in China. Color depth indicates affected provinces, and dots indicate root rot pathogens based on China Academic Journals full-text database (CNKI), Web of sciences (WoS), and other websites.

In China, root rot was first recognized by Yao (1989) in Xingjiang and Gansu provinces. Large areas of alfalfa were found to be affected by root rot. Presently, alfalfa root rot has been reported in 11 provinces, with more in the northwest (Xinjiang, Gansu), northeast (Heilongjiang, Jilin), and north region (Inner Mongolia; Figure 1). China’s pastoral areas are concentrated in these regions, particularly the northwest and north regions, where extensive livestock-raising is the leading agricultural enterprise. A range of forages, including alfalfa, is grown in these regions. Root rot is likely to occur in alfalfa in Qinghai, Liaoning, and Tibet, as reported on other legumes (Zhimin, 1996). Still, we could not find any published report on alfalfa root rot in these provinces (Figure 1). Furthermore, there are no published reports of root rot in China’s southern and south regions. These regions are agricultural areas where limited forages are sown. Many pathogens have been shown to have varying degrees of direct involvement in causing root disease. *Fusarium* spp. and *R. solani* are predominant and widely distributed root rot pathogens and have been most frequently isolated and described (Yao, 1989; Gang et al., 1996; Li, 2003; Li et al., 2009; Liu et al., 2016; Wang et al., 2016; Cong et al., 2016a; Jiang et al., 2021). Also, these pathogens are serious root rot pathogens in various economically important crops, including grain and forage legumes worldwide (Barbetti and MacNish, 1984; Barbetti et al., 2007; Ajayi-Oyetunde and Bradley, 2016, 2018). The distribution of *Fusarium* spp. associated with alfalfa root rot in the northeast, north, and northwest region of China is relatively well understood compared to other pathogens, as shown in Table 1. Studies showed that environmental factors, mainly moisture, and temperature, affect root rot (Barbetti et al., 2007). Rainfall in these regions is erratic, resulting in the production of chlamydospores and sclerotia that enable *Fusarium* spp. and *R. solani* to survive in prolonged dry periods in these regions. The same distribution trend of these fungi was also noted in the semi-arid, arid, and subhumid regions of the world (Barbetti, 1983; Smiley and Yan, 2009). For example, in Australia, *Fusarium* spp. is highly distributed in the dry regions where rainfall is erratic. Accordingly, the fungus produces chlamydospores, enabling them to survive more in the dry period than other pathogens (Summerell et al., 2011). In recent years, oomycetes such as *Pythium* spp. and *Phytophthora* spp. are causing severe root rot in the irrigated areas of Gansu and Sichuan provinces (Yinghua et al., 2019; Zhang et al., 2020). In addition, *Macrophomina phaseolina, Phoma* spp., *Paraphoma* spp. and *Microdochium tabacinum* have also been recovered from root rot in Gansu province and Inner Mongolia (Kaur et al., 2012; Wang et al., 2015a; Lakhran et al., 2018; Cao et al., 2020; Marquez et al., 2021; Zhao et al., 2021). These fungi in combination with *Fusarium* spp., *R. solani* and oomycetes resulting in severe root rot (Min-Quan et al., 2003; Huang et al., 2013; Wen et al., 2015b; Cong et al., 2016b; Zhang et al., 2020; Wang et al., 2020b; Fang et al., 2021). In contrast to northwest and north, the northeast region (Heilongjiang, Jilin) is relatively fertile well-developed agriculture and has plenty of water. From the northeast region, *Bipolaris sorokiniana* and *Alternaria alternata* in addition to above mentioned pathogens have also been reported (Li et al., 2019; Jiang et al., 2021).

**Table 1:** Distribution of *Fusarium* spp. in the alfalfa-growing areas of China.

Region	Province	Location^*^	*Fox*	*Fso*	*Fac*	*Feq*	*Fse*	*Fav*	*Fpr*	*Ftr*	*Fsp*	*Fch*	*Fcu*	*F*in
Northwest	Gansu	Dingxi	+		+		+							
		Huanxian	+		+	+	+	+						
		Jiuquan	+	+										
		Qingyang	+	+	+		+	+						
		Wuwei										+		
		Zhangye	+	+										
		Jinchang												
	Xinjiang	Hutubi	+	+	+	+		+			+			
		Altay												
		Urumqi												
		Yining												
	Shaanxi	Yulin			+									
		Dingbian			+									
North China	Inner Mongolia	Ar Horqin	+							+				
		Ordos	+	+										
		Chifeng	+								+			
		Hohhot	+	+									+	
		Linhe		+						+				
		Tongliao												
	Hebei	Cangzhou	+	+		+			+					
		Langfang				+				+				
		Huanghua	+	+	+	+			+	+				
		Zhangjiakou	+		+				+	+				
		Unknown												+
		Xuanhua	+		+	+				+				
	Shanxi	Gaoyang		+					+					
Northeast	Heilongjiang	Daqing	+	+	+	+				+				
		Xiangfang	+	+	+	+				+				
		Shuangcheng	+	+	+	+				+				
		Acheng	+	+	+	+				+				
		Lanxi	+	+	+	+				+				
		Qiqihar	+	+										
		Zhaodong	+	+	+	+				+				
		Harbin												
		Unknown												
	Jilin	Unknown	+			+								

*Fox: F. oxysporum; Fso: F. solani; Fac: F. acuminatum; Feq: F. equisti; Fse: F. semitectum; Fav: F. avenaceum; Fpr: F. proliferatum; Ftr: F. tricinctum; Fsp: F. sporotrichioides; Fch: F. chlamydosporum: Fcu: F. culmorum*; Fin: *F. incarnatum*. + = Isolation from that location. ^*^Location: city/country, References: Wang et al. (1996), Liu and Yu (2006), Cong et al. (2017), and Yinghua et al. (2019).

## Fungal and oomycetes pathogens

**Figure 2:**
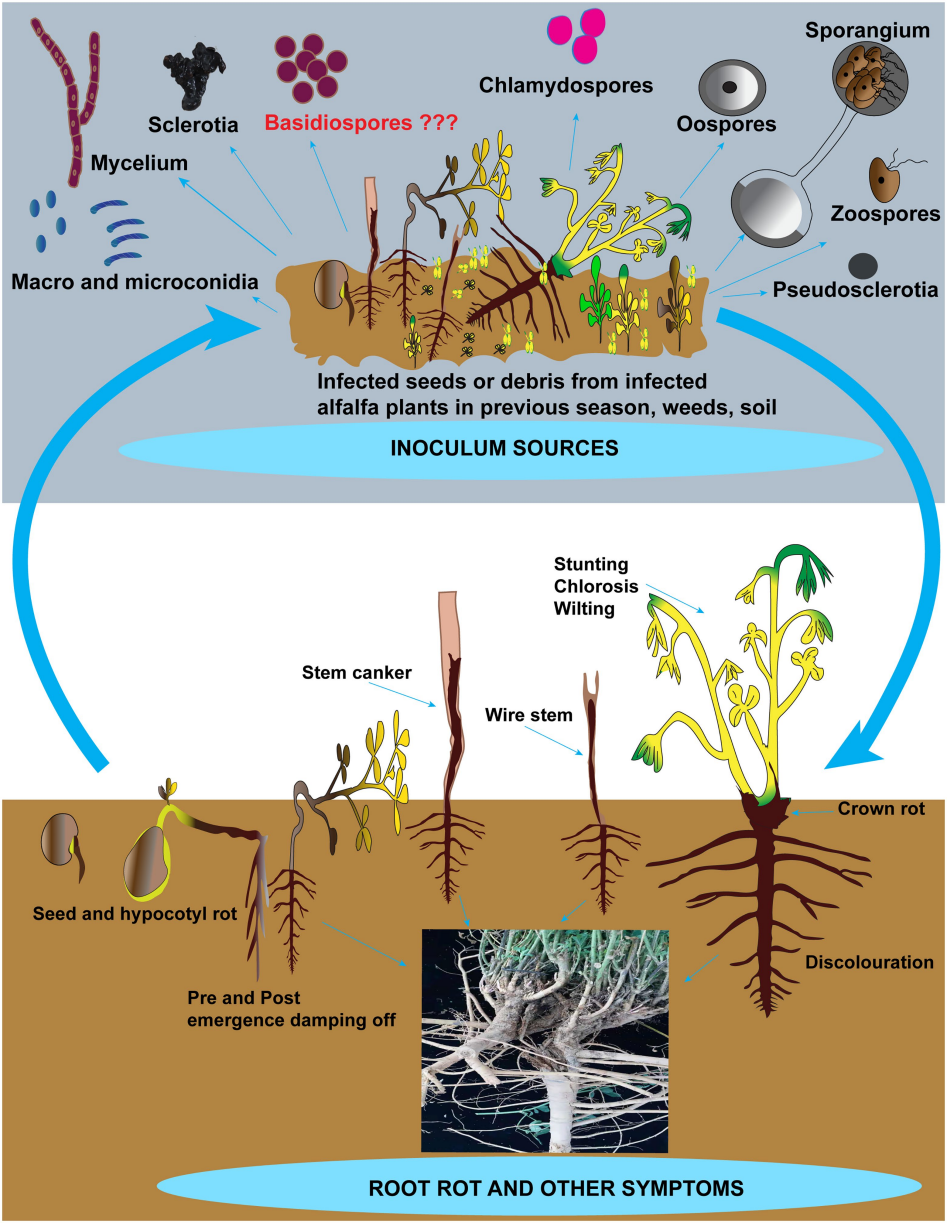
Schematic representation of alfalfa root rot and other symptoms and inoculum sources. Inoculum sources include zoospores, oospores, sporangia, conidia, chlamydospores, sclerotia, mycelia, and basidiospores (not reported). Besides root rot, other symptoms include damping-off, seed and hypocotyl rots, discoloration on roots, crown rot, stunting, chlorosis, wilting, and wire stem.

Most root rot pathogens are soil-borne and can survive for many years by producing resilient structures such as sclerotia and chlamydospores. Figure 2 depicts the inoculum sources, including the resilient structures and symptoms in addition to root rot caused by these pathogens on alfalfa. Recent reports suggest that these pathogens can also be seed-borne (Kong et al., 2018b). However, there is no report on the relationship between the disease incidence of root rot and pathogen-associated with alfalfa seeds. Hence seed transmission seems unimportant in the etiology of root rot of alfalfa in China. Barbetti (1983) removed fungi from the clover seeds and found no influence on the severity of root rot diseases after sowing (Barbetti, 1983). Among these pathogens, *Fusarium* spp. and *R. solani* are accountable for incalculable losses to alfalfa compared to other fungi. The status of these pathogens involved in root rot in China and abroad, biological characteristics and disease cycles are addressed further in the below sections. Other fungi such as *Bipolaris sorokiniana*, *Paraphoma* spp. and *Phoma* spp. that contribute to root rot in alfalfa but are not causing significant losses are shown in Table 2 (de Gruyter et al., 2009, 2010; Moslemi et al., 2018; Jiang et al., 2021).

**Table 2:** Records of pathogens other than *Fusarium* spp. in the alfalfa-growing regions of China.

Region	Province	Location^*^	*Bs*	*Ph.c*	*P.c*	*Pr*	*Pm*	*Rs*	*Aa*	*Cg*	*Mp*	*Mt*	*Pa*	*Ps*	*Hs*
Northwest	Gansu	Dingxi						+							
		Huanxian						+				+			
		Jiuquan			+			+							
		Qingyang						+							
		Wuwei			+										
		Zhangye			+			+			+		+	+	+
		Jinchang		+				+							
	Xinjiang	Hutubi						+							
		Altay						+							
		Urumqi						+							
		Yining						+							
	Shaanxi	Yulin													
		Dingbian													
North China	Inner Mongolia	Ar Horqin													
		Ordos													
		Chifeng				+									
		Hohhot													
		Linhe													
		Tongliao										+			
	Hebei	Cangzhou													
		Langfang													
		Huanghua													
		Zhangjiakou													
	Henan	Yuzhong													
	Shanxi	Gaoyang													
Northeast	Heilongjiang	Daqing					+		+	+					
		Qiqihar													
		Zhaodong					+		+	+					
		Harbin	+												
		Unknown					+		+	+		+			
	Jilin	Unknown					+								

*Bs: Bipolaris sorokiniana, Ph.c: Phytophthora cactorum, P.c: Pythium coloratum, P.r: Paraphoma radicina; Pm: Phoma medicaginis; Rs: R. solani; Aa: A. alternata; Cg: Chaetomium globosum: Mp: Macrophomina phaseolina; Mt: Microdochium tabacinum; Pa: Phoma alfalfa; Ps: Phoma solani; Hs: Helminthosporium solani*. + = Isolation from that location. ^*^Location: city/country, References: Yuan et al. (2003), Wang et al. (2015a, 2020b), Wen et al. (2015b), Fan and Li (2017), Fan et al. (2018), Cao et al. (2020), Zhang et al. (2021), and Zhao et al. (2021).

### *Fusarium* spp.

*Fusarium* is a cosmopolitan genus that includes filamentous ascomycetes fungi (Sordariomycetes: Hypocreales: Nectriaceae). Link (1809) was the first to describe and identify the genus (Link, 1809). The genus is exceptionally complicated, and its taxonomy has always been contentious due to polyphyletic grouping. Currently, between 100 and 500 species are reported globally (Summerell et al., 2010). The genus includes pathogens that cause severe disease of plants, endophytes, saprophytes and produce several mycotoxins and/or phytotoxins. These mycotoxins and/or phytotoxins can render alfalfa unfit for animal feeding, and some of them may act as virulence factors in enhancing root rot disease (Nedelnik and Repkova, 1997). According to a recent investigation, toxins, including trichothecenes, zearalenone, and fumonisins are not only involved in disease pathogenesis but can also cause human and animal poisoning in China. Besides, these toxins severely affect the germination of alfalfa seeds (Kong et al., 2018a). Fusarium diseases collectively include wilts, rots, blights, and cankers of many horticultural, field, ornamental, forage, and forest crops in natural and agricultural ecosystems (Coleman, 2016). *Fusarium* root rot caused by *Fusarium* spp. is harmful in all growing stages of alfalfa, consequently reducing nitrogen fixation ability and longevity, and productivity (Kong et al., 2018a).

#### Status of *Fusarium* root rot in China and abroad

*Fusarium* spp. root rot is the most common root disease of many crops, including alfalfa globally (Table 3). This disease has considerably affected alfalfa in the United States, Canada, New Zealand, Australia, Italy, and India. Countries like Egypt and Japan also reported more than 50% prevalence rates in some areas (Samac et al., 2013). In China, *Fusarium* root rot is endemic in the north (Inner Mongolia), northeast (Heilongjiang, Jilin), and northwest (Xinjiang, Gansu, Qinghai) regions. The most affected provinces in these regions are Xinjiang, Gansu, Inner Mongolia and Heilongjiang (Fang et al., 2019, 2021). The disease hinders the establishment of the alfalfa stand, reduces yield and forage quality, and shortens the plant’s lifespan (Gang et al., 1996; Wang et al., 2005). In the northwest provinces, i.e., Xingjiang and Gansu, a 60% death rate of alfalfa was recorded. In the northeast region of China, i.e., in Jilin and Heilongjiang provinces, the disease becomes severe in August, with an incidence rate of 20 to 40% and a peak rate of around 92%. Similarly, in north China, i.e., in Inner Mongolia and Hebei, the disease becomes severe from August to October, with an incidence rate of 15 to 30% (Shouyan et al., 2011; Chen et al., 2015b; Cong et al., 2018; Wang et al., 2020a; Jiang et al., 2021). Besides, *Fusarium* spp. and *R. solani* co-infection or mixed infections increased root rot disease severity and decreased alfalfa growth and biomass allocation (Li et al., 2009; Wei et al., 2016; Fang et al., 2021). Among the *Fusarium* spp. *F. oxysporum* is the most damaging to alfalfa production in China (Fang et al., 2021; Table 1). *F. oxysporum* is also one of the top ten most economically important fungal pathogens, with over 100 formae speciales (f. spp.) based on host specificity (Edel-Hermann and Lecomte, 2019). Furthermore, *F. oxysporum* is the host-specific pathogen of alfalfa and other *Medicago* spp. (Batnini et al., 2020).

**Table 3:** Reports of plants affected by *Fusarium* root rot.

Pathogens	Plants	Major disease	Distribution	References
*F. roseum*	Alfalfa	Root and crown rot	Canada	McKenzie and Davidson, 1975
*F. avenaceum, F. solani, F. oxysporum, F. acuminatum, F. sambucinum, and F. avenaceum*	Alfalfa	Root and crown rot	USA	Snyder et al., 1991; Salter et al., 1994
*F. solani, F. oxysporum, F. roseum, F. tricinctum*	Alfalfa	Root and crown rot	Canada	Richard et al., 1980; Bugbee and Campbell, 1990
*F. solani, F. tricinctum, F. avenaceum, F. oxysporum, F. roseum*	Alfalfa	Root and crown rot	Canada	Hwang and Flores, 1987; Hwang et al., 1989
*F. avenaceum, F. solani, F. oxysporum*	Alfalfa	Root rot	Worldwide	Miller-Garvin and Viands, 1994
*F. avenaceum*	Clover, Pea	Root rot	Worldwide	You et al., 2005; Eranthodi et al., 2020
*F. culmorum*	Wheat	Root rot and head blight	Worldwide	Erginbas-Orakci et al., 2018
*F. graminearum*	Maize	Earmold and root rot	Worldwide	Löffler et al., 2010
*F. graminearum*	Soybean	Pod blight and root rot	Worldwide	Zhang et al., 2010
*F. pseudograminearum*	Barley, wheat	Crown rot	Wheat and barley growing regions	Liu et al., 2012
*F. solani f sp. batatas*	Sweet Potato	Storage rot	China	da Silva and Clark, 2013
*F. solani f. sp. phaseoli*	Bean	Root rot	Bean growing region except Australia	Hagerty et al., 2015
*F. solani f. sp. pisi*	Pea	Root rot	Worldwide	Porter et al., 2014
*F. verticillioides*	Maize	Root and ear rot	Worldwide	Miedaner et al., 2010
*F. semitectum*	Alfalfa	Root rot	Italy	Zaccardelli et al., 2006

#### Biological characteristics, disease cycle, and damages to alfalfa

**Figure 3:**
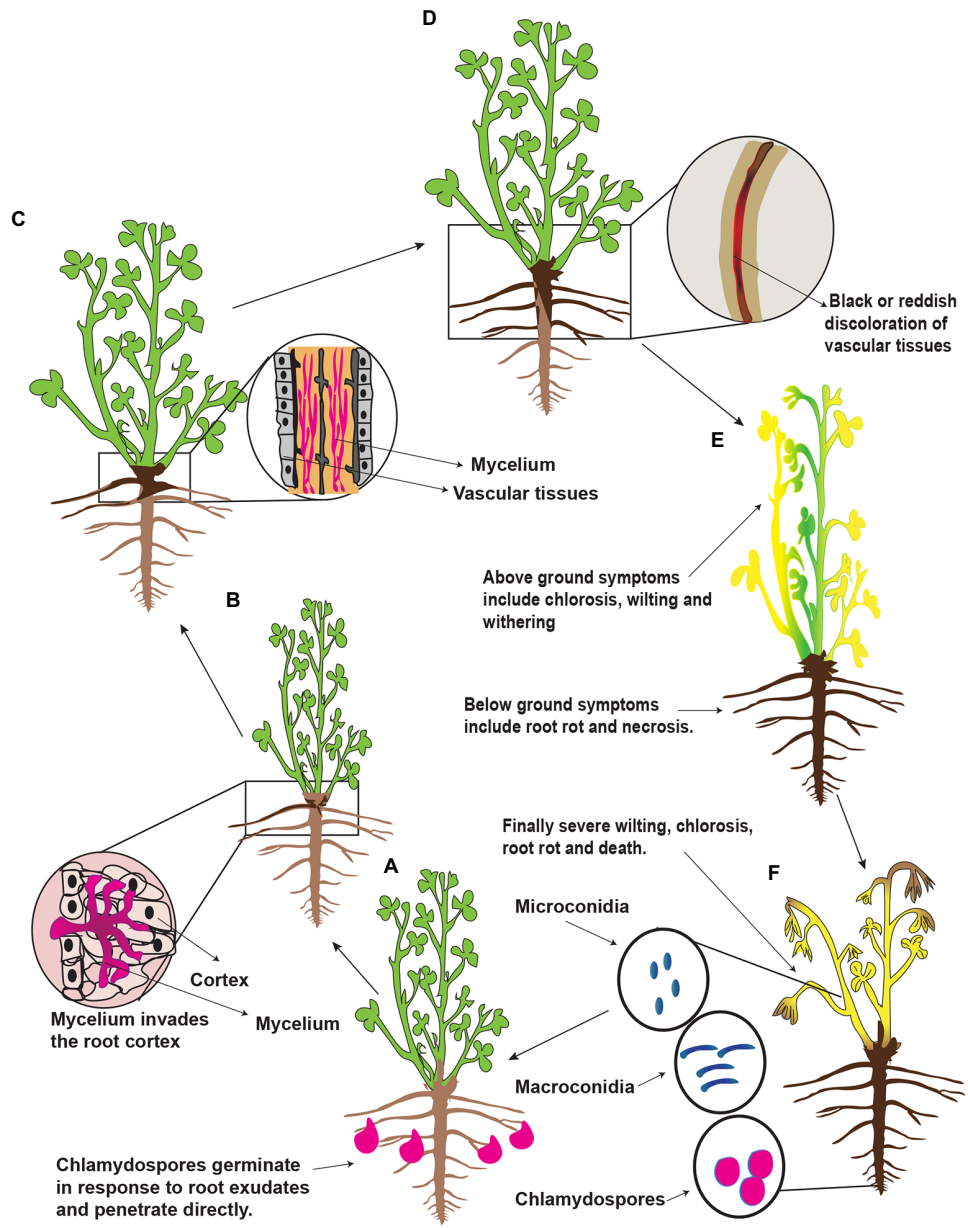
Schematic representation of the presumed disease cycle of *Fusarium* root rot in China. **(A)** Alfalfa plant secrete root exudates and in response to exudates *Fusarium* spp. spores (chlamydospores) germinate and produce infection hypha to penetrate the root epidermis at the root tip. **(B)** The hypha proliferates in the root cortex and enters into the vascular vessels, i.e., xylem vessels. **(C)** In the vessels, it grows excessively and causes a blockage; as a result, brown discoloration occurs. **(D)** First symptoms appear at the base of the stem, and then the symptoms progress upward; as a result, the young leaves withered. **(E)** Partial chlorosis or complete chlorosis is observed mainly on the mature leaves. **(F)** Finally, the whole alfalfa plants wilt because of severe root rot followed by death. Fungal spores such as microconidia, macroconidia, and chlamydospores form dead alfalfa plant tissues and remain dispersed in the soil.

*Fusarium* spp. overwinter on plant debris/residues, seeds, and soils in the form of spores (microconidia and macroconidia), mycelial fragments, and chlamydospores (Cong et al., 2016a,b). The chlamydospores are more durable dormant structures and are regarded as the principal form in which *Fusarium* spp. survives in the soil for decades. These are enlarged thick-walled vegetative cells and are considered adaptations for survival during unfavorable environmental conditions such as prolonged dry periods (Fang et al., 2019). Chlamydospores usually formed on and in roots showing root symptoms and sometimes present when rot symptoms were not evident (Leath and Kendall, 1978). Once the conditions become favorable, the chlamydospores germinate in response to alfalfa’s root exudates, which contain a wide array of organic compounds, including sugars and amino acids (Fang et al., 2019). A schematic representation of the presumed disease cycle of *Fusarium* root rot is shown in Figure 3. Infection hyphae from chlamydospores and/or mycelium penetrate the epidermis of rootlets, taproots, and stem base directly or through wounds or injuries and reach to root cortex. Consequently, brown to black necrotic patches are formed around the roots. Then the hypha reaches vascular vessels (xylem) and blocks the water. Finally, the stele of the roots decay, and the collar and the center of the root become hollow and lateral roots rot in large quantities. On the surface of roots, necrotic spots are formed, the tangential face of roots becomes brown or black, and reddish-brown or dark brown stripes/lesions are formed. The roots’ internal and external portions show red-brown discolorations (Berg et al., 2017; Wang et al., 2020a; Li et al., 2021a). In the later stage of the disease, the plants become weak and can be easily removed from the earth. Severely infected plants showed stress-type foliar symptoms such as chlorosis, brown and reddish-brown foliage color, withering of individual twigs, or the whole plant wilting. These symptoms are frequently exacerbated by infrequent rainfall and cool soil temperatures. Besides, *Fusarium* spp. break cold resistance of alfalfa (Richard et al., 1982) predispose alfalfa to other pathogens. In addition, the taproot is the first root that penetrates the upper soil layer; if it is rotted, then the greatest reduction in plant size can be expected. However, the rotting of lateral roots had little effect on plant size because most plants regenerate new lateral roots to compensate for the loss of the lateral root (Pegg and Parry, 1983; Barbetti and MacNish, 1984). Furthermore, the infected plants dispersed among apparently healthy plants or the affected areas may occur in distinct patches. As a result, alfalfa plantations are sparse in a few years. Once alfalfa root rot occurs in a large area, it will seriously reduce alfalfa production and even need to be replanted (Kong et al., 2018a). The foliar symptoms of root rot in alfalfa vary in the northwest, northeast and north of China, most probably because of particular environmental conditions. Micro and macroconidia are secondary sources of infection to alfalfa and can survive on the surface of contaminated plants and spread to adjacent plants. Hence, chlamydospores play a vital role in the occurrence and circulation of root rot disease. The number and survival of the chlamydospores directly affect the occurrence of the disease and its degree of harm. Furthermore, *Fusarium* spp. cause severe damage to those alfalfa plants which are already weakened or injured by other abiotic factors, i.e., stress factors. For example low soil temperature and soil compaction predispose alfalfa to *Fusarium* root rot (Li et al., 2021a). Further*, Fusarium* spp. can infect any developmental stage of alfalfa; therefore, alfalfa cannot find a chance to escape from the *Fusarium* spp. Consequently, the alfalfa’s life, the nitrogen-retaining capacity, and the crop’s quality reduce, resulting in the loss of alfalfa’s processing value. Besides, the soluble sugar content of the infected alfalfa plants reduces, resulting in reduced regenerative capacity, slow growth, and reduction of yield. The gradual appearance of sparse plots and plants seriously affects alfalfa production. Further, they can survive in the soil for a long time and accumulate year after year, resulting in a decrease in alfalfa’s resistance to disease, resulting in a longer planting age (Fang et al., 2019). A previous report showed that chlamydospores could survive in the soil for up to 30 years, meaning that infected land cannot be used to replant alfalfa (Fones et al., 2017). Recent reports suggest that *Fusarium* spp. also infect the alfalfa seeds. For example, 150 *Fusarium* strains from rotting alfalfa and induced pathogenicity on the alfalfa seeds (Kong et al., 2018a). All the isolated *Fusarium* strains induced pathogenicity on the germinated alfalfa seeds with varying pathogenic intensities. However, there is still a lack of any relationship between disease incidence of root rot and pathogen-associated with seed. A study showed that removing pathogens from the seeds had no influence on root rot severity after sowing (Barbetti, 1983).

### Rhizoctonia solani

**Figure 4:**
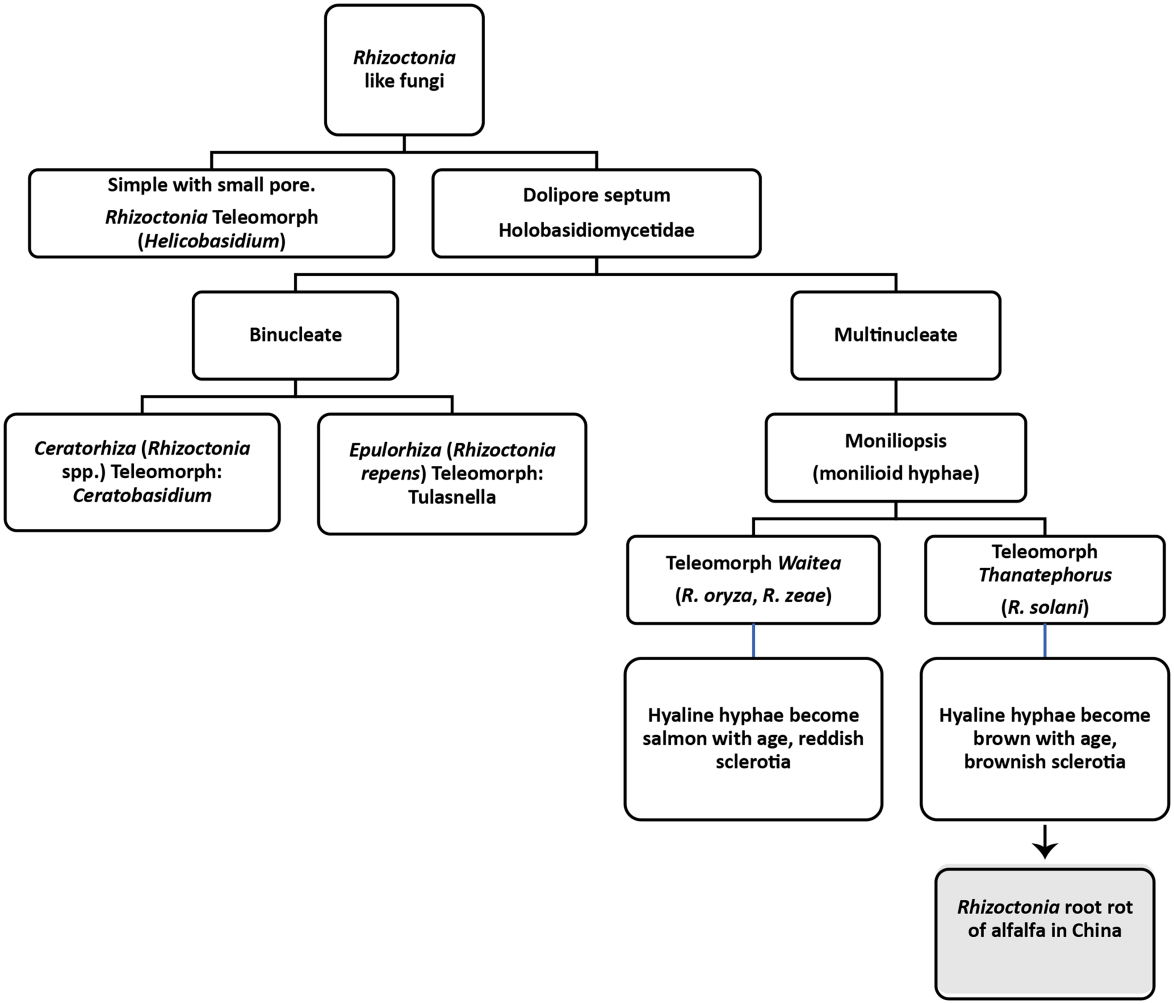
Classification of *Rhizoctonia* like fungi based on Moore (1987).

*Rhizoctonia* genus was first introduced in 1815 by de Candolle for an unknown fungus severely infecting alfalfa and saffron (De Candole, 1815). According to de Candolle, the basic characteristics of the genus are the presence of sclerotia, mycelia emanating from sclerotia and the association of mycelia with the roots of plants. Moore et al. divided *Rhizoctonia* like fungi into four genera based on teleomorph, as shown in Figure 4 (Moore, 1987). Later classification of *Rhizoctonia* has been revised into three main teleomorphic genera (Stalpers and Andersen, 1996; Sneh et al., 2013). One is multinucleated *Thanatephorus cucumeris* (Frank) Donk (anamorph: *R. solani*). The other one is a bi-nucleate fungal genus known as *Ceratobasidium* (anamorph: *Ceratorhiza*) and the third one is also a multinucleated genus known as *Waitea* (anamorph: *Rhizoctonia zeae*; Vilgalys and Cubeta, 1994; Andersen, 1996). All these genera lack clamp connections; however, differences have been found in the moniloid hyphae, sclerotia and dolipore septa (Stalpers and Andersen, 1996). *Rhizoctonia solani* (synonym: *Thanatephorus cucumeris*), the most studied and important necrotrophic fungus causing roots rots and other plant diseases, is of most significant interest to plant pathologists (Ajayi-Oyetunde and Bradley, 2016). It was first observed on diseased potato tubers by Kuhn in 1858 (Kühn, 1858). It damages 200 hosts, including cereals, vegetables, agricultural trees, horticultural trees, forest trees, weeds, ornamentals, and forage crops. The important features of *R. solani* comprise septate hyphae, multinucleate cells in young hyphae, the brown coloration of mature hyphae, right-angled hyphal branching, constriction at the point of branching, dolipore septa that allows unrestricted cell-to-cell movement of cytoplasm, mitochondria and nuclei, production of monilioid cells, and sclerotia of uniform texture. Clamp connections, rhizomorphs, conidia and sexual states other than *T. cucumeris* and hyphal pigmentations other than brownish mature hyphae have never been observed. This fungus cause root rots, hypocotyl rot, crown rot, stem rot, limb rot, pod rot, stem canker, black scurf, seedling blight, and pre-and post-emergence damping-off in different plants (Stalpers and Andersen, 1996).

#### Status of *Rhizoctonia solani* root rot in China and abroad

Root rot caused by *R. solani* was first reported by Fatemi (1972), who isolated fungus from the rotting roots of alfalfa in Fars province, Iran (Fatemi, 1972). The disease can now be found in many countries of the world including China. The amount of damage it produces in alfalfa varies, but the losses can be significant, even dramatic. Besides, there is considerable diversity in the cultural and colony morphology, host range, molecular and biochemical markers, pathogenicity, virulence, nutritional requirements among isolates of *R. solani*. Therefore *R. solani* is considered a species complex consisting of reproductively isolated and non-interbreeding populations. The isolates readily undergo hyphal and cytoplasmic fusion, and exchanging nuclei are grouped in the same anastomosis groups (AGs). In contrast, isolates that fail to achieve hyphal and cytoplasmic fusion and nuclear exchange is considered members of different AGs. *R. solani* isolates have been divided into 14 AGs (AG-1 to AG-13) and an AG-bridging isolate AG-B1 based on hyphal anastomosis reactions, cultural morphology, pathogenicity/virulence, and DNA homology (Ogoshi, 1987). These AGs are further divided into subgroups based on anastomosis frequency, physiological and morphological characteristics, biomolecular, biochemical, genetic and DNA homology features (Blanco et al., 2018). AGs such as AG-1, AG-2, and AG-4 are mainly associated with stem and root rot diseases in dicots, while AG-8 is associated with root rots in monocots. In general, AG-1, AG-2, AG-3, and AG-4 cause severe diseases in plants globally, whereas the remaining AGs are less harmful and have limited geographic distribution (Eken and Demirci, 2003; Li et al., 2009; Zhang et al., 2021). AGs responsible for severe root rot of alfalfa across the world are; AG-1 and AG-4 in the United States (Vincelli and Herr, 1992), AG-1, -2, -3, -4, -5, and 10 in Turkey (Eken and Demirci, 2003), AG-4 in Iran (Balali and Kowsari, 2004), AG-1 to -10 in Saudi Arabia (Alkherb et al., 1997) and AG-11, -8, and -6 in Australia (Anderson et al., 2013; Oladzad et al., 2019). The anastomosis group of *R. solani* causing rot diseases of economically important crops, including alfalfa, are highlighted in Table 4. In China, root rot of alfalfa is caused by AG-1, AG-2, AG-4, and AG-5; however, so far, no binucleate *Rhizoctonia* spp. have been reported to cause root rot of alfalfa in China (Tables 2, 4). In addition, the occurrence frequency of AG-2 and AG-5 was higher than AG-1 and AG-4. Additionally, the pathogenicity of each anastomosis group was also significantly different and AG-2 had the highest pathogenicity on alfalfa (Li et al., 2009). In 2015, the pathogenicity of six isolates of *R. solani* was checked on 14 alfalfa varieties. All the isolates resulted in lower germination rates. Besides, many seedlings died before emergence and a large percentage of seedlings died after emergence due to root rot (Guo et al., 2019). Recently, a study was conducted to determine whether any host resistance to *R. solani* exists among the alfalfa varieties. A considerable variation in disease responses among the alfalfa varieties was observed, with the range of disease indices of shoots from 23 to 94%, roots from 31 to 98%, and reductions in dry weight of shoots from 35 to 96% and roots from 2 to 99% (Zhang et al., 2021).

**Table 4:** Economically important plants including alfalfa affected by anastomosis groups (AGs) of *Rhizoctonia solani* root rots.

Anastomosis groups (AGs)	Plants	Major disease	Distribution	References
AGs 1, 2, 4 &5	Alfalfa	Root rot	China	Li et al., 2009
AGs 5 & 8	Barley	Root rot	Worldwide	Rush et al., 1994; Carlucci et al., 2012
AGs 2, 4 & 5	Bean	Root rot	Worldwide	Oladzad et al., 2019
AGs 1, 2 & 4	Carrot	Crown and brace root root	Worldwide	Punja, 2005
AGs 2, 4 & 5	Faba bean	Root rot	Worldwide	Assunção et al., 2011
AGs 1	Lettuce	Bottom rot	Germany	Dijst and Schneider, 1996
AGs 2 and 4	Oilseed rape	Root rot and damping-off	Worldwide	Verma, 1996
AGs 4	Pea	Root rot	Worldwide	Hwang et al., 2007
AGs 1,2 and 4	Soybean	Root rot	Worldwide	Rahman et al., 2018
AGs 2	Sugar beet	Root rot	Worldwide	Nagendran et al., 2009
AGs 3 and 4	Tomato	Foot and root rot	Worldwide	Nikraftar et al., 2013
AGs 8	Cereals	Root rot, Bare patch	Worldwide	Neate, 1989; Okubara et al., 2016
AGs 1 & 4	Alfalfa	Root rot	USA	Vincelli and Herr, 1992
AGs 1, 2, 3, 4, 5 & 10	Alfalfa	Root rot	Turkey	Eken and Demirci, 2003
AG-4	Alfalfa	Root rot	Iran	Balali and Kowsari, 2004
AGs 1–9	Alfalfa	Root and crown rot, stem canker	Saudi Arabia	Alkherb et al., 1997
AGs 11, 8 and 6	Alfalfa	Root and hypocotyl rot, root canker	Australia	Anderson et al., 2013
AG-4	Alfalfa	Seed rot	USA	Gónzalez et al., 2016
AG-3	Tomato	Leaf blight and root rot	Japan	Misawa et al., 2020
AG-1	Clovers	Summer blight and root rot	China	Bai et al., 2014
^*^Unk	Cow pea and beans	Root rot	Oman	Al-Jaradi et al., 2018
AGs 1–13	Potatoes	Stem rot	USA	Woodhall et al., 2012
AG-4	Chickpea	Root rot	Turkey	Basbagci et al., 2019; Basbagci and Dolar, 2020
AGs 1–3	Various legumes	Wet root rot and webblight	India	Dubey et al., 2014
AGs 2 & 4	Lupin	Stem and root rot	Canada	Zhou et al., 2009
AGs 1, 2 & 4	Common bean	Web blight and root rot	Central and South America, Turkey	Godoy-Lutz et al., 2008; Kilicoglu and Ozkoc, 2010; Spedaletti et al., 2016
AG-4	Pea	Root rot	USA	Mathew et al., 2011
AGs 2, 4 & 5	Canola and wheat	Root rot	Canada	Broders et al., 2014
AGs 1, 2, 3, 4, 7 & 11	Soybean	Seedling and root diseases	USA, Canada, Brazil	Fenille et al., 2003; Ajayi-Oyetunde and Bradley, 2016
AG-2	Onions	Root rot	USA	Coleman et al., 1997; Brown et al., 2020
AGs 2 & 3	Tobacco	Root rot and leaf spot	Worldwide	Gonzalez et al., 2011
AGs 4, 5 & 6	Strawberry	Root rot	USA and South Africa	Botha et al., 2003; Sharon et al., 2007

* Unk, unknown.

#### Biological characteristics, disease cycle, and damage to alfalfa

**Figure 5:**
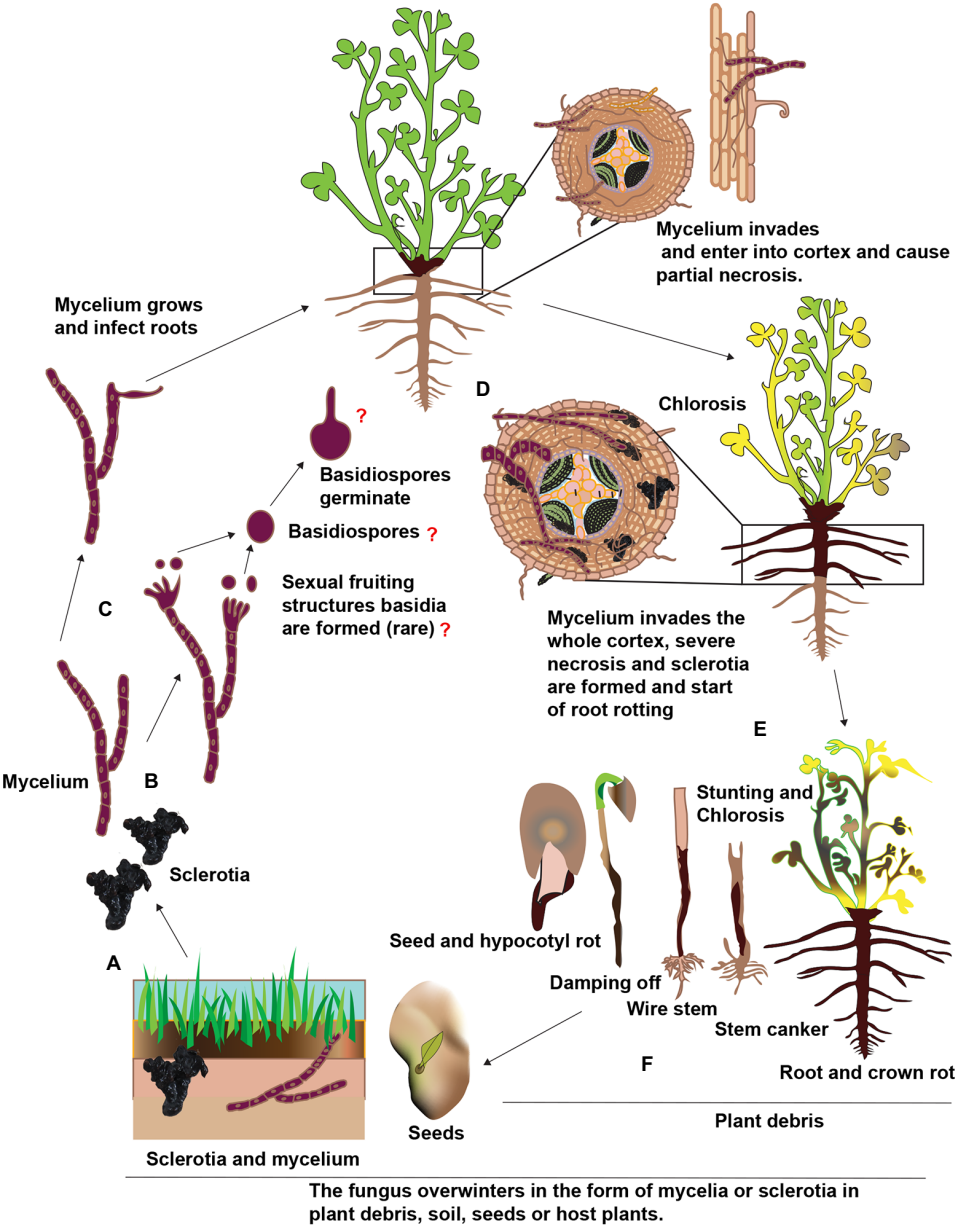
Schematic representation of the presumed disease cycle of *Rhophitulus solani* root rot in China. **(A)**, The fungus overwinters in the plant debris and seeds in the form of mycelium and in the soil as sclerotia and mycelium. **(B,C)** The young hyphae germinate and develop under favorable conditions, sexual fruiting structures basidia and basidiospores are rare. **(D)** The mycelium penetrates roots near the soil line and colonized in inter and intracellular spaces. **(E)** The mycelium proliferates further in the cortex ultimately results in necrosis and sclerotia are formed in and on infected tissues and disintegration and acute rotting of roots. **(F)** Above ground symptoms, include chlorosis, blights, stunting and finally death, the fungus also infects seeds and seedlings and also causing damping-off.

*Rhizoctonia solani* doesn’t produce vegetative or asexual spores, e.g., conidia. The role of sexual spores, e.g., basidiospores, as an inoculum source for the alfalfa root rot disease is unknown. In addition, the *R. solani* is a facultative parasite and can easily compete with other soil born saprophytes. In order to survive in the soil, it develops sclerotia, a long-lasting structures or propagules (Williamson-Benavides and Dhingra, 2021). These nutrient-independent propagules are formed from the undifferentiated hyphae or monilioid cells. When the conditions become favorable, sclerotia germinate and mycelia are formed. These mycelia are attracted to alfalfa roots in response to the root exudates. A schematic representation of the presumed disease cycle of *Rhizoctonia* root rot is shown in Figure 5. Upon reaching to roots, hypha grows along with the epidermal cells and forms appressoria to penetrate the alfalfa tissues by infection pegs. Hence, sclerotia are considered as the primary inoculum of root rot. The pathogen is also considered necrotrophic and produces extracellular hydrolytic enzymes to kill its host in advance of colonization (Ajayi-Oyetunde and Bradley, 2018). Unfortunately, there is little information regarding the pathogenicity of *R. solani* on alfalfa, whether the discoloration and necrotic of the alfalfa roots are the results of certain toxic or enzymes secreted by *R. solani*. If the environmental conditions are favorable, the sclerotia germinate and form hyphae, enter the root cortex, and continuously grow inside and on the surface of alfalfa roots. Thereby, longitudinal blackish lesions appear on the roots and at advanced stages, the roots become decay and rot. Also, the crown area becomes dark brown or black (Zhang et al., 2021). Foliar symptoms include yellow or reddish color or wilt leaves. *R. solani* also infect seeds and usually, infected seeds don’t germinate and if they germinate, the seedlings are killed before or after emergence. Sclerotia forms, again, thereby completing the disease cycle and can remain viable for several years under harsh environmental conditions such as temperature, starvation, desiccation, chemicals, and severe radiation. According to previous research, the sclerotia of *R. solani* can remain viable in soil without a host for 8 to 10 years and as the primary inoculum. In addition, the pathogen also can survive in the form of mycelium in the plant debris. The mycelia and sclerotia are spread by irrigation water, rain and floods to other alfalfa fields (Garibaldi et al., 2021).

### Oomycetes

Oomycetes (syn. Peronosporomycete), often known as “water molds,” are a group of hundreds of organisms (between 600 and 1,500 species; Dick et al., 1999). They were assumed to be closely related to the kingdom Fungi for a long time because of their similar ecological and morphological traits (Harper et al., 2005). However, they are now thought to be phylogenetically distinct from fungi, with diatoms, chromophyte algae, and other heterokont protists being their closest relatives. Therefore, they have been placed in a separate kingdom, Stramenopiles, consisting of the most devastating plant pathogen. They cause seedling blights, damping-off, foliar blights, downy mildew, and root rot. In contrast to fungi, their cells walls contain cellulose and have tubular mitochondrial cristae and are vegetative diploid (Van West et al., 2003). They have the potential to survive both in aquatic and terrestrial environments. Oomycetes such as *Aphanomyces* spp., *Pythium* spp., and *Phytophthora* spp. are causing severe root rot of alfalfa growing regions in the world (Table 5). They survive in the soil in the form of rigid, resistant structures called oospores. Oospores germinate directly by producing germ tubes in response to chemical signals from the alfalfa host or proliferate as sporangia (Harper et al., 2005). Zoospores with heterokont flagella (one tinsel and one whiplash) inside the sporangia are formed. Zoospores are released from sporangia and swim through water-filled soil pores with the help of flagella. Previous reports suggest that soil moisture significantly affect the incidence and severity of root rot caused by oomycetes (Erwin, 1954; Faris and Sabo, 1981; Gray et al., 1983). Also, the disease becomes more severe when the soil remains wet for ten days or longer (Kuan and Erwin, 1980). Water saturation predisposes alfalfa to oomycetes by increasing root damage and exudation of nutrients like amino acids and sugars that boost the chemotactic attraction of zoospores to roots (Gray et al., 1983). Once a zoospore reaches the root surface of alfalfa, it loses both flagella, encysts and germinate by forming a germ tube. Hyphae derived from the germ tube directly penetrate the root epidermis and colonize roots. The hyphae then differentiate into antheridia and oogonia within the roots, forming oospores. Oospores survive in the soil for many years in the absence of alfalfa. Both *Pythium* spp. and *Phytophthora* spp. are a severe problem only in the irrigated and/or flood irrigated alfalfa-growing areas. *Aphanomyces* spp. have been recognized as a severe root rot pathogen of legumes in several American states and other legumes-growing regions of the world, notably in Europe (Gaulin et al., 2007). We could not find a published report of root rot of alfalfa caused by *Aphanomyces* spp. in China. However, there are reports on root rot of other legumes caused by *Aphanomyces* spp. (Zhimin, 1996). In many studies, oomycetes were found to form pathogens complexes with *R. solani* and *Fusarium* spp., and inflicting severe damage to alfalfa roots causing root rot and damping-off, resulting in reduced yields, decreased winter survival, and shortened stand life (Hancock, 1983; Berg et al., 2017).

**Table 5:** Reports of plants affected by oomycetes root rots.

Pathogens	Plants	Major disease	Distribution	References
*Aphanomyces cochlioides*	Sugar beet	Root rot and damping-off	Across all sugar beet plantations	Taguchi et al., 2010
*Aphanomyces euteiches*	Alfalfa, bean, pea	Root rot and damping-off	Asia, Europe, Oceania, North America	Hagerty et al., 2015
*Phytophthora citrophthora*	Citrus	Root rot and fruit rot	Worldwide	Park et al., 2008
*Phytophthora nicotianae*	Citrus	Crown, root, and fruit rot	Worldwide	Sakupwanya et al., 2018
*Phytophthora cactorum*	Apple, strawberry	Root, crown rot and damping-off	Worldwide	Carisse and Khanizadeh, 2006; Eikemo et al., 2010
*Phytophthora cinnamomi*	Avocado	Root and heart rot	Worldwide	Douhan et al., 2011
*Phytophthora fragariae*	Raspberry, strawberry	Red stele or red core root rot.	Asia, Australia, New zealand, Europe, North America	Park et al., 2008
*Phytophthora sojae*	Soybean	Root and stem rot	Canada, Australia, USA, Chila, China, Korea, New Zealand	Park et al., 2008
*Phytophthora capsici*	Pepper	Fruit, stem, and root rot	Worldwide	Kim et al., 2008
*Phytophthora medicaginis*	Alfalfa, chickpea, soybean	Root rot	Greenhouse and field settings	Dale and Irwin, 1991; Park et al., 2008; Vandemark and Barker, 2010
*Pythium ultimum*	Mostly vegetables	Root and seed rot	Worldwide	Bruno and Griffiths, 2004; Bates et al., 2008
*Pythium irregulare*	Clover, Soybean	Root rot and damping-off	Greenhouse and field settings	Mao et al., 1998; You et al., 2005; Ellis et al., 2013
*Pythium aphanidermatum*	Mostly vegetables	Root and stem rot, damping-off	Greenhouse and field settings	Fattahi et al., 2011; Aliyu and Balogun, 2012

#### *Phytophthora* spp.

The genus *Phytophthora* consists of more than 100 species, and the majority of them are aggressive plant pathogens that cause extensive losses in agricultural, horticultural and forage crops (Cai et al., 2021). *Phytophthora* means “plant destroyer,” a term coined in the 19th century when they destroyed potato fields of Ireland, causing the Great Irish Famine (Yang et al., 2017). Erwin first described *Phytophthora* root rot of alfalfa in California, United States. He observed the disease as causing severe root rot in less than three years old alfalfa plants in high rainfall, heavily irrigated, poorly drained soils (Erwin, 1954). Initially, he named the pathogen *P. cryptogea;* however, later, the pathogen was classified as *P. megasperma*. *Phytophthora* root rot is prevalent in almost all alfalfa-growing regions of the United States, Canada, and Australia (Kuan and Erwin, 1980; Faris and Sabo, 1981; Rogers, 1981; Gray et al., 1983, 2004). Among the *Phytophthora* spp. i.e., *P. infestans*, *P. megasperma*, *P. citrophthora*, *P. cactorum*, *P. cinnamomic*, *P. fragariae*, *P. sojae*, *P. capsici*, *P. nicotianae*, and *P. medicaginis* are causing severe root rot including forages worldwide (Steinmetz et al., 2020). In China, *P. cactorum* has been reported to cause severe root rot. In 2018, 2 years old alfalfa plants in Jinchang, Gansu province, were severely infected by *P. cactorum*. There were red to dark-brown discolorations in the taproots. Affected plants have wilting shoots with decaying and rotting taproots and lateral roots. Besides sporangia, chlamydospores, and oospores were also recovered (Cai et al., 2021). The symptoms of alfalfa root rot were similar to symptoms on alfalfa crops caused by *Phytophthora* in other countries (Erwin, 1954; Marks and Mitchell, 1970; Rogers, 1981). The disease cycle begins with zoospores which move freely in water and contact with the tips of rootlets. The lesions on the roots become yellowish and then brownish and later turn to dark brown or black, often with halo margins. The size and type of root lesions depend on the duration of wet soil conditions, alfalfa genotypes, or both. In severe conditions, lateral and tap roots are rotted, and foliar symptoms such as yellowing, stunting and wilting appear (Marks and Mitchell, 1970).

#### *Pythium* spp.

The genus *Pythium* contains more than 200 described species, and at least 10–15 species are causing damping-off and root rots in various agricultural, horticultural and forage crops (Beckerman, 2010; Berg et al., 2017; Wang et al., 2020a). Symptoms on alfalfa caused by *Pythium* are like *Phytophthora* that cause root rots; however, root tips become necrotic in the early infection. Furthermore, the entire primary roots become black and rotting moves upward to the stem (Zhang et al., 2020). Four important *Pythium* spp. such as *P. ultimum*, *P. irregulare*, *P. aphanidermatum*, and *P. myriotylum* have been reported from alfalfa fields and are the most common root rot causing pathogens in the world (Williamson-Benavides and Dhingra, 2021). During 2017 and 2019, 30 to 80% of alfalfa plants in Gansu province stunted, wilted, and dried. Likewise, irregular brown necrotic lesions were observed on the taproots. In addition, the lateral roots showed brown discoloration and were poorly developed, necrotic, and rotted. Morphological characters of sporangia, oogonia and antheridia, were identified. Morphological characters and molecular identification suggested that the pathogen was *P. coloratum* (Zhang et al., 2020).

## Effects of environmental factors on root rot

The occurrence, severity, and prevalence of root rot are affected by primary infection sources, management practices, environmental factors, and alfalfa cultivars’ ability to resist disease (Williamson-Benavides and Dhingra, 2021; Fahad et al., 2021a,b). Among environmental factors, such as soil moisture and soil temperature, play a significant role in the occurrence, severity and prevalence of root rot (Jat and Ahir, 2013). Root rot of alfalfa caused by oomycetes is not generally severe in alfalfa growing regions of China. Oomycetes root rot is higher in locations with excessive rainfall, heavily irrigated or poorly drained soil or flood irrigated (Liang et al., 2006; Cai et al., 2021). For example, in the southwest region (Sichuan province), which has comparative more rainfall than northwest (Xingjiang and Gansu) and north (Inner Mongolia) of China, oomycetes are causing severe rot of legume crops. Besides, the soil of this region is wetter and less dry than the northwest, northeast and north region of China. Oomycetes produce oospores which germinate into hyphae under high soil moisture conditions (generally in compacted soil). Furthermore, oomycetes such as *Phytophthora* spp. and *Pythium* spp. are causing the most severe root rotting over the range of temperatures 17–23°C. Moreover, oomycetes root rot develops in alfalfa when the soil remains excessively moist for about ten days or longer (Karppinen et al., 2020). Many researchers postulated that excessive soil moisture cause lack of oxygen that predispose alfalfa to oomycetes root rot (Fahad et al., 2021c,d). Hence the incidence of root rot caused by oomycetes increased with the increase in soil water content, and the lower soil moisture could reduce the development of root rot disease (Fahad et al., 2020; Cai et al., 2021). However, oomycetes become most aggressive against alfalfa when a cool, wet spring is followed by an early, warm, dry summer (Yinghua et al., 2019). Wong et al. (1984) checked how soil moisture interacted with the pathogenicity of *Pythium* spp., *Phytophthora* spp. *Fusarium* spp. and *Rhizoctonia* spp. both alone or in combination. These fungi and their combination caused root disease over the range of soil moisture conditions. The most severe root rotting occurred at 65% water holding capacity and less at 45% (Wong et al., 1984). It indicates that high soil moisture favor the growth of oomycete pathogens. Soil moisture and soil temperature also influences the *Phoma* and *Paraphomra* root rot of alfalfa. For example, *Paraphoma* and *Phoma* root rots of alfalfa are favored by moderate soil temperatures (15–21°C) and soil moistures (60–70%; Cao et al., 2020; Deb et al., 2020). In 2014, seven cultivars of alfalfa were grown in seven different fields in the Chifeng county in Inner Mongolia. The variability of disease incidence of *Paraphoma* root rot was extremely high and the potential reason was that soil moisture content at these fields varied due to the uneven terrains (Cao et al., 2020). Furthermore, the peak period of root rot also varies according to changes in humidity and temperature in the alfalfa-growing regions of China. For example, in the north region of China, the peak period for root rot disease is the first week of August, whereas the peak period for the disease in the southwest region is in mid-September (Cao et al., 2008). *Fusarium* spp. and *R. solani* are causing more severe root rot in the drier soils and lower rainfall regions such as northeast, northwest and north of China. However, both fungi can cause severe root rot at high soil temperature (24–32°C) and soil moisture (70–80%; Ajayi-Oyetunde and Bradley, 2018). In addition, the number of infections’ propagules of these fungi varies seasonally. After January, it began to rise, peaking in May and June, and then began to drop from July through December (Wong et al., 1984). Similarly, each anastomosis groups (AGs) of *R. solani* require certain temperature to cause root rot (Kousik et al., 1995). Furthermore, *Fusarium* spp. and *R. solani* also infect alfalfa at cold temperatures (15–18°C), which slows down alfalfa seedlings’ growth (Coleman, 2016; Wang et al., 2020a). Winter survival of alfalfa depends on the accumulations of food reserves in the roots and crown. However, both *Fusarium* spp. and *R. solani* reduce the cold resistance of alfalfa and predispose alfalfa to winterkill by affecting the accumulation of food reserves (Hwang et al., 1989). Research reports on other legumes showed that even changes in the soil pH affected the alfalfa survival and root rot disease severity caused by *Fusarium* spp. *Phythium* spp. and *R. solani*. These pathogens responded to pH differently, and alfalfa resistance to individual pathogens also varied depending on the amount of lime added (Barbetti, 1990). Furthermore, these fungi infect alfalfa mostly in spring and temperature ranging from 15 to 25°C was found to be optimum for infection of alfalfa (Yuan et al., 2003). Alfalfa root rot caused by *Bipolaris* spp. is favored in dry and warm soil and a temperature range from 15 to 25°C. Also, when alfalfa plants are grown under stress conditions such as warm and less moist soil, the root rot caused by *Bipolaris* spp. become severe (Acharya et al., 2011). Moreover, the erratic weather conditions herald a rise in mean temperatures as well as other natural disasters like droughts, floods, and storms. These circumstances are anticipated to put alfalfa under constant stress, which is anticipated to encourage the pathogens that cause root rot to become more active (Fahad et al., 2021e,f). Furthermore, soil of alfalfa growing regions of China is nutrient-poor, compact, and not very fertile and generally lacks phosphorus, resulting in significantly reduced nutrient levels (Wang et al., 2021). Previous studies showed that nutrient-poor and leached soils impose nutrition stress on crops; as a result, natural resistance to root rot disease reduced (Graham, 1983). By adding appropriate nutrients to alfalfa-growing regions, the root rot disease severity can be reduced because plant nutrients promote growth and enhance disease tolerance to root rot. Especially phosphate should be added to alfalfa fields, which can enhance host resistance by stimulating the production of phytoalexins against the virulence factors of root rot pathogen (Barbetti et al., 2007). Besides the activities of antagonists that restrict root rot, pathogens and other soil microflora can also be affected by soil nutrients. Hence, soil nutrients influence disease severity by changing root physiology and host resistance and by influencing the interaction between the host and the pathogen and/or antagonist, each of which can be influenced independently by the availability of nutrients. In China, the interaction of soil nutrients with root rot pathogens of alfalfa is currently unknown, however, with greater knowledge, root rot can be managed by altering soil nutrients. In addition, temperature and moisture also affect mycelia growth, sporulation, germ tube growth, and spore germinations. For example, the appropriate temperature range for *Fusarium* spp. to produce spores is from 25 to 32°C and the appropriate temperature range for *R. solani* to produce vegetative structures such as mycelia is 20–30°C and to produce overwintering structure such as sclerotia is 25°C. Similarly, sporangia of *P. megasperma* germinated indirectly by releasing zoospores in the flooded soil at 8–24°C (Pfender et al., 1976). Furthermore, each pathogen in the root rot complex has its specific optimal developmental conditions. For example, the optimum temperatures for the mycelia growth of *F. equiseti* and *F. proliferatum* were 20 and 28°C, respectively, (Kong et al., 2018b). Even the developmental conditions vary for each strain of same species. For example, the optimum temperatures for the mycelial growth of different *F. oxysporum* strains, D19-2 and B1-63 were 26 and 28°C, respectively (Kong et al., 2018b) Besides, soil pH also influences the developmental phases of root rot pathogens. For example, the optimum pH for the mycelial growth of *F. equiseti* and *F. proliferatum* was 6 and 10, while the pH for sporulation and spore germination was 8.0 and 7.0, respectively (Kong et al., 2018b). Similarly, alternating light and dark are important for mycelia growth, and light is important for sporulation. Spores do not germinate when the relative humidity is lower than 75% (Pan et al., 2015). As previously stated, among the environmental factors, soil moisture, and soil temperature significantly impact the root rot disease of alfalfa. More research is required to understand how soil moisture, soil temperature, and other environmental factors such as pH affect root rot of alfalfa in natural pastures.

## Management strategies

Many strategies have been evaluated to manage the root rot in recent years. They are explained below.

### Cultural approach

The cultural approach makes conditions unfavorable to the root rot pathogens to reduce root rot disease severity (Sumner, 1994). The conditions for the root rot pathogens can be made unfavorable for root rot pathogen in many ways, such as soil pH and soil nutrients adjustment, fallowing, composting, timely grazing, biofumigation, sanitation, mixed cropping, intercropping and crop rotation. Fallowing is to keep the area fallow for several years before cultivation of alfalfa, however, most of the root rot pathogens are soil borne, the inoculum remains even after fallowing (Barbetti and MacNish, 1984). Bio-fumigation and sanitation in combination can also effectively reduce the survival structure of root rot pathogen such as microsclerotia and sclerotia in the soil (Wang et al., 2014). However, production losses from fallowing and bio-fumigation cannot be bearable for farmers. Inoculating seeds with rhizobia or BCAs can also reduce the root rot severity of other legume crops. For example, *Rhizobium trifolii* significantly reduced root rot of clover caused by *F. avenaceum* in glasshouse studies (Wong et al., 1984). However, if the soil is acidic or contains a high concentration of available aluminum, in that case it negatively affects the rhizobia growth and survival and interferes with the legume-rhizobia symbiosis by affecting rhizobia attachment to roots (Tabares-da Rosa et al., 2019). As outlined above, the soil of pastoral areas is poor nutrients deficient in phosphates. Therefore, the effect of soil nutrition and soil pH is probably so significant to manage root rot. The productivity of alfalfa can be improved through the application of phosphate. However, this may enhance soil acidity in many areas making conditions less favorable for the growth of alfalfa. Besides, the addition of lime to manipulate soil pH should also be further investigated as lime influences alfalfa and root rot pathogens. In a previous study, pathogen complexes associated with root rot such as *Fusarium* spp., *Pythium* spp., and *R. solani* responded differently to the addition of lime (Barbetti, 1990). It indicates that these pathogen complexes can be broken by altering soil pH resulting reduction of root rot severity. Furthermore, dense planting should be avoided to reduce the disease spread. Also, avoid planting too deep because it takes more time to emerge from the soil, increasing the chances of getting infected by soil pathogens (Hwang et al., 2002). In addition, leftovers, stubbles, infected plants, debris and weeds should be burned to reduce the disease inoculum. In addition, the application of green organic manures, farmyard manure and organic fertilizers, including composts, green manures and animal manures, reduce the root rots and promote the growth of beneficial soil microbes significantly (Wiggins and Kinkel, 2005). Moreover, the amount and time of application of inorganic fertilizers are also crucial for disease development (Summers, 1998). The application of nitrogenous fertilizers should be discouraged because they make the plants succulent, and pathogens get more chances to infect the plants (Stone et al., 2003). Alfalfa seedlings are more susceptible to *Pythium* spp. and *R. solani* following seed germination; hence high-quality seeds which can rapidly germinate should be sown. Excessive irrigation, compaction and poor drainage of alfalfa fields should be avoided because these conditions favor oomycetes. Besides, judicious application of fungicides in combination with appropriate cultural practices can significantly reduce the disease severity. Soil compaction in the alfalfa fields is also a major problem. Continuous cropping of alfalfa, application of inorganic fertilizers and reduction in the use of green or animal manures cause soil compaction (Stone et al., 2003). Increased soil compaction affects aeration, porosity, and water retention capacity while increasing bulk density. Plant growth, biomass, and yield are reduced as a result, the incidence and severity of root rot diseases rise (Williamson-Benavides and Dhingra, 2021). Multiyear continuous cropping of alfalfa allows root rot pathogens to continue their uninterrupted disease cycle, resulting in their perpetuation and multiplication. However, rotating with unrelated crops or related crops of differential disease susceptibility such as clovers, cereals and soybeans, many root rot pathogens having a narrow host range can be controlled successfully (Curl, 1963). However, crop rotations are not effective against soil-borne pathogens, especially against *Fusarium* spp. and *R. solani*. These fungi produce survival structures such as chlamydospores and sclerotia and survive for several years. Besides, most of the pathogens on alfalfa are common to other rotational legumes and/or crop species. For example, *R. solani* is a common pathogen of alfalfa and rice. Similarly, *Pythium* spp. and *Fusarium* spp. can cross infect between pasture legumes such as alfalfa and clovers and/or crop species such as barley, wheat and oat (Sivasithamparam, 1993). However, host-mediated selection occurs in the pathogen isolates and if that is, then root rot diseases can be limited to a certain extent. Besides, grazing and harvesting could be timely to reduce the impact of root rot, especially at the seedling stage when plants are more vulnerable to root rot (Barbetti, 1990). For example, in Inner Mongolia (North China), alfalfa is harvested in autumn in some areas. Studies showed that autumn harvest reduces organic residues in the roots; as a result, plants become weak to face winter and predispose to *Fusarium* root rot (Couture et al., 2002). In conclusion, cultural practices are ineffective against root rot in susceptible alfalfa cultivars. Root rot can only be managed effectively when resistant cultivars are grown besides appropriate cultivation and cultural practices and judicious use of fungicides.

### Breeding, transgenic, gene silencing, and editing approach

#### Breeding approach

Currently, 77 alfalfa cultivars have been registered in China. Among these, 36 cultivars were bred through breeding programs, 17 were introduced from other countries, five domesticated from wild ecotypes, and 19 were collected from the regional/breeding programs (Zhang et al., 2021). These commercial alfalfa cultivars acquired by farmers in China are created and sold by seed corporations and marketed as pests resistant. However, so far, the breeders could not develop a resistant cultivar against alfalfa root rot. There are seven main reasons which limit the selection of alfalfa-resistant cultivars: (i) Alfalfa is a perennial plant that is primarily cross-pollinated, and several factors influence its self-fertility; (ii) Alfalfa is autotetraploid; the breeding and selection factors are different from diploid plant species; (iii) Genetic complexity of root rot pathogens. There are varieties with resistance to a single pathogen; however, developing varieties with resistance to multiple pathogens which cause alfalfa root rot is challenging; (iv) Pathogens vary in different environmental conditions in the saturated soil; for example, oomycetes become dominant and in the dried soil *Fusarium* spp. and *R. solani* becomes dominant; (v) Alfalfa cultivars cannot adapt to different environmental conditions; (vi) Pathogen evolution is speedy compared to breeding, which takes 20–30 years to discover resistant markers and new cultivars; and (vii) Lack of understanding of resistance mechanisms in alfalfa roots to root rot pathogens. Therefore, there is a delay in utilizing breeding approaches to develop root rot-resistant cultivars compared to other crops. In China, selective breeding, cross-breeding, male-sterile line breeding, space breeding, biotechnology-assisted breeding, transgenic technology, and molecular marker technology are all employed to improve resistance in cultivars against many diseases, but not against root rot. For example, researchers have tried to develop disease-resistant varieties using molecular marker technology, i.e., Random Amplified Polymorphism (RAPD) technique and Bulked Segregation Analysis (BSA) were used to study molecular markers linked to resistance genes against a brown spot disease in five *Medicago* species (Gui and Yuan, 2002). Furthermore, R-gene mediated resistance is race-specific, and resistance to root rot is quantitatively inherited. However, alfalfa resistance can be improved by stacking or pyramiding major R genes/QTLs for multiple pathogens associated with root rot (Fuchs, 2017). We could not find a published report on the pyramiding R genes/QTLs (quantitative trait loci) against root rot in China. It indicates that studies regarding QTLs mapping of alfalfa to uncover genetic architectures related to root rot are in preliminary stages. In other countries, pyramiding R genes/QTLs have achieved resistance to root rot. For example, QTLs were mapped in various plants that contributed to high-level resistance against root rot caused by *Phytophthora* and *Pythium* spp. Though QTL mapping has been a powerful technique in identifying genomic regions associated with root rot resistance traits in bi-parental populations (Ramalingam et al., 2020). However, QTL mapping has limitations because QTL cannot detect natural variations in diverse genetic backgrounds due to low allelic diversity and recombination rates in bi-parental populations. To overcome these limitations, genome-wide association study (GWAS) is widely used to evaluate broader genetic diversity and inquire greater quantities of recombination due to the evolutionary history of natural populations. For example, a candidate gene encoding F-box protein was identified in alfalfa, using GWAS, as a negative regulator of resistance to root rot caused by *Aphanomyces* spp. (Bonhomme et al., 2014). There is also a high-resolution NGS SNP data developed in the *Medicago truncatula* HapMap Project^1^ which involved sequencing of 288 *Medicago* accessions by Illumina technology (Stanton-Geddes et al., 2013). Recently, to mitigate GWAS and QTL mapping limitations, these two QTL analysis approaches are being combined, providing complementary, robust, and vigorous assays to uncover the genetic basis underlying complex traits. For example, the genetic architecture of *Aphanomyces* root rot resistance in lentils has been dissected by linking QTL Mapping and GWAS (Ma et al., 2020). Recently scientists are trying to examine the genetic architecture of root rot disease resistance in other legume crops by QTL Mapping and GWAS. These studies will highlight the accumulation of favorable haplotypes in the most resistant accessions against root rot disease (Ma et al., 2020). Most research in China has concentrated on screening germplasm accessions and commercial cultivars in greenhouses and fields only against strains of *Fusarium* spp. and *R. solani*, which could serve as possible sources of resistance. For example, in a field screening assay, 20 alfalfa cultivars were inoculated with several *F. oxysporum* isolates, four *F. acuminatum* isolates, and five *F. semitectum* isolates. Only seven cultivars showed resistance to *Fusarium* spp., including Verla, Derful, Ameristand 201, Caoyuan 2, Sitel Algongum, Tumu 2, and Gannong 2. The rest of the cultivars were susceptible to *Fusarium* spp. (Quan, 2003). Thirty alfalfa cultivars were tested in another screening assay against three *Fusarium* species: *F. solani*, *F. semitectum*, and *F. camptoceras*. Only three varieties, “Delilande,” “Xinjiangdaye” and “Muxuwang” were resistant to *F. semitectum* with disease index 18.11, 19.93 and 19.96, respectively. With a disease index of 25.30 to 39.95, seven varieties were found to be disease tolerant. The disease index ranged from 40.55 to 59.56 for eighteen varieties. The remaining varieties were susceptible, with disease index ranging from 61.7 to 68.6. These findings indicate that no alfalfa variety is immune or resistant to alfalfa root rot (Ding et al., 2011). In a recent study, about 68 alfalfa varieties were screened for resistance against *R. solani* (Zhang et al., 2021). Among these, three varieties (Gannong 9, Trifecta and Common), originating from three different countries, exhibited a high level of resistance, with disease indices of shoots and roots and reductions in dry weight of shoots and roots being all ≤ 40%. In addition, five varieties (7%) showed resistance, 15 (22%) were moderately resistant, and the remaining ones exhibited susceptibility. In addition to screening alfalfa varieties against root rot pathogens, intensive efforts have also been dedicated to elucidating the defensive response to the pathogen invasion. For example, in a study, alfalfa plants were inoculated with *Fusarium* spp. In the resistant varieties of alfalfa, enzyme phenylalanine aminase (PAL) was more active than in the sensitive varieties whereas other defensive enzymes such as superoxide dismutase (SOD), peroxidase (POD), polyphenol oxidase (PPO) were more active in the susceptible varieties than the resistant varieties (Fang et al., 2003; Quan et al., 2003). These studies offer valuable resistance sources for breeding programs to develop alfalfa varieties with improved resistance to root rot pathogens and for facilitating the identification of molecular mechanisms underlying the resistant varieties to this pathogen. Unfortunately, most the studies regarding alfalfa disease resistance use a single strain or multiple strains of a fungal pathogen; it’s impossible to tell whether the cultivars chosen are disease-resistant in general to the diversity of pathogens in the field conditions.

#### Transgenic approaches

Transgenic approaches are widely employed to manage root rot disease of other crops. In a study, an alfalfa seed antibacterial peptide-encoding gene (*alfAFP*), was fused to the C-terminal of the rice chitinase-encoding gene and transferred into tobacco. In transgenic tobacco plants, the recombinant protein improved resistance to *F. solani*. Even 30 days after being inoculated with *F. solani*, transgenic lines did not show root rot (Azam et al., 2018). Pathogenesis-related proteins (PRs) are widely distributed in plants, including alfalfa, and are essential in defense responses. Their expression is regulated by specific hormone signaling pathways (Li et al., 2021b). These PRs proteins have different functions, i.e., production of hydrolytic enzymes such as glucanases and chitinases, which lysis cell wall components (Sunpapao and Pornsuriya, 2016), thaumatin-like and osmotin-like proteins which weaken cell walls and permeabilized plasma membranes (Rather et al., 2015), antimicrobial peptides (Nawrot et al., 2014) and RNAse activities to degrade pathogens RNA (Tang et al., 2019). Hence, applying reverse genetics technology involving gene overexpression and gene silencing (e.g., RNAi) has enabled the rapid functional characterization of *PR* genes. For example, when the *PR5* gene was overexpressed in the *M. truncatula*, the resistance responses such as Abscisic acid (ABA) production and signaling and reactive oxygen species (ROS) were high after inoculation with *A. euteiches*. Besides, disease resistance against *A. euteiches* was linked to the lignification and production of a small GTPase (20-O-methylisoliquiritigenin) which regulated ROS (Badis et al., 2015). A recent study showed Recombinant PnPR10-3 functions as an RNase *in vitro* exhibited strong antifungal effects on *Fusarium* species (*F. oxysporum*, *F. solani*, and *F. verticillioides*; Tang et al., 2019). On the other hand, reverse genetics has been found to overcome the limits of traditional breeding approaches. However, producing transgenic cultivars resistant to numerous pathogens that cause root rot remains difficult. Fortunately, the recent advent of transcriptomics and next-generation sequencing technologies offers the potential to identify genes involved in root rot resistance on a broader scale (Song et al., 2016).

#### Genes silencing and editing approach

Currently, host-induced gene silencing (HIGS) is being used, whereby the host produces double-stranded RNA molecules (dsRNA) that target pathogen genes and are processed into short interfering RNA molecules (siRNAs; Perez et al., 2021). Pathogens procure these siRNAs upon infection; consequently, their target genes are silenced. HIGS has been successfully applied against viral, pests, parasitic plants, and fungi. The main advantage of this method is to silence the pathogen genes without introducing new proteins into food and food products. To date, HIGS has been successfully used against mildews, rusts, and wilting diseases of agriculturally important crops. Recent discoveries of gene-editing technology have made it possible to target pathogenesis-related genes. CRISPR/Cas9 editing has been recently used to inhibit the infection caused by pathogens (Gupta et al., 2021). For example, the pathogen *R. solani* activates the *OsSWEET11* sugar transporter in the infected rice tissues to acquire sugar molecules for its nutrition. However, when the sugar transporter, *OsSWEET11* was knock-out using CRISPR-Cas9, it was found that rice crops became less susceptible to rice sheath blight disease as compared to *OsSWEET11* overexpressing and wild-type plants (Gao et al., 2018b). These modern approaches may help to deploy resistance against root rot rapidly. However, no transgenic anti-root rot alfalfa cultivars have been developed in China and even globally. Besides, these approaches face public and political distrust (Fones et al., 2020).

### Biological approach

Concerns regarding the chemicals have recently grown among the general public. Chemicals are wreaking havoc on the ecosystem. Researchers are increasingly focused on biocontrol agents (BCAs), and there have been several success stories about the use of biological agents thus far. Competition for nutrients or space, antibiosis, induce host resistance and lytic enzyme production are the recognized mechanisms by which BCAs control root rot (Fravel, 2005). BCAs are becoming more popular, and however, in the case of forage crops, their use is still limited in China. The reason might be survival, growth, adaptation, and establishment of biological agents in the fragile pastures ecosystems of China is challenging. To date, only a few fungal and bacterial BCAs have been used to manage root rot. So far, among the fungal BCAs, saprophytic fungi *Trichoderma* spp. and arbuscular mycorrhizal fungi *Glomus* spp. have been used against root rot. *T. harzianum*, *T. koningii* and *Glomus mosseae* were tested against *Fusarium* spp. i.e., *F. solani*, *F. oxysporum*, *F. semidaricum*, and *F. solani* and *Microdochium tabacinum*. Besides significantly reducing root rot, they also enhanced alfalfa growth and nutrient uptake (Li et al., 2018; Zeng et al., 2019). In another experiment, *Glomus mosseae* and *Rhizobium* (*Sinorhizobium medicae*) combined effects on the root rot caused by *Microdochium tabacinum* have significantly reduced root rot besides helping alfalfa to uptake water and nutrients, specifically phosphorus (Gao et al., 2018a). Bacterial BCAs, *Bacillus* spp., *Pseudomonas* spp., and *Actinomycetes* are also widely used to manage alfalfa root rot (Xiao et al., 2002; Knezevic et al., 2021). In 2009, about 91 actinomycetes were isolated from 10 soil samples in Chifeng Inner Mongolia through the gradient dilution separation method. Most of them have significantly reduced root rot caused by *Fusarium solani*, *F. oxysoporum* and *F. avenaceum* (Wang et al., 2010). In another study, *Bacillus subtilis* subsp. *spizizenii* (MB29) was evaluated against *F. semitectum*. The strain effectively reduced the mycelia growth of *F. semitectum*. Furthermore, *in vivo* test, MB29 significantly reduced root rot, producing a disease control efficiency of 43.41% (Wen et al., 2015a). In another study, cultural filtrates of *Ochrobactrum intermedium* strain (I-5) significantly reduced the spore production, germination, and mycelia growth of *F. tricinctum*. In addition, a 10% filtrate of the strain reduced root rot by >73% in repeated experiments. Besides, the strain enhanced invertase, urease, cellulose, and neutral phosphatase activity in the rhizosphere soil and reduced root rot-related soil quality damage. Also promoted the growth of alfalfa without causing apparent damage to plants (Li et al., 2021a,c). Currently, antagonists which are endophytes have also been used to manage root rot. Endophytes are more protected than free-living (rhizospheric) (Lugtenberg et al., 2016) BCAs because they inhabit the internal tissues of plants without causing disease, forming a close symbiotic relationship. For example, seeds are the reservoir of endophytes that protect plants from root rot. They can infiltrate the host systems without exposing them to pathogens. Plant health and vigor, as well as root persistence through rotation, can be improved with the use of endophytes (White et al., 2019). Chen et al. (2015a) isolated 363 strains of endophytes from alfalfa fields in Hebei, Inner Mongolia, and Ningxia provinces of China. These strains include fungi, bacteria, and actinomycetes. Among these strains, three endophytic bacterial strains, e.g., NA NX51R-5, NA NX90R-8, and NA NM1S-1, showed strong biocontrol capability with > 50% effectiveness against *F. oxysporum* under in vitro and pot experiments. The strains NA NM1S-1 and NA NX51R-5 were identified as *Bacillus* spp. while the strain NA NX90R-8 was *Pseudomonas* spp.. There are also some drawbacks to employing BCAs against root rot pathogens. In most cases, a single BCAs is employed to fight against a single pathogen (Raupach and Kloepper, 1998). This may sometimes account for inconsistent field performances even though their efficacy was quite good under controlled conditions (Nicot, 2011). This variability of efficacy is generally due to environmental variations (changing soil temperatures and moisture) in the field, a lack of ecological competence (such as the ability to survive and colonize), intrinsic characteristics of the antagonistic microbe (such as variability in the production of required metabolites or enzymes), and/or an unstable quality of the formula (Bardin et al., 2015). In addition, efficacy may be reduced due to diversity in sensitivity of pathogens to biocontrol agents with the presence of less sensitive isolates in the natural populations of plant pathogens (Fravel, 2005). Hence single BCA is not active in all environmental conditions especially against the pathogen complexes of root rot. More attention should be paid to the application of a mixture of BCAs that can better cope with environmental changes during the growing season and defend against pathogen complexes associated with root rot. Increased genetic diversity of BCAs may allow them to stay longer in the rhizosphere and utilize a wide array of antagonistic activities against root rot pathogens.

### Chemicals, phytochemicals, and elicitor approach

Though many management approaches have been attempted to combat root rot, chemical control still remains the primary method for managing root rot. Fungicides are used as seed treatment and soil application to protect the alfalfa from root rot (Min et al., 2002). For example, Fludioxonil, hexaconazole, tebuconazole, propiconazole, difenoconazole, vitavax, carbendazim, captan, metalaxyl, thiophanate methyl, shenqinmycin mefenoxam and mancozeb alone or in combinations were applied as seed treatment and/or as a soil application to control root rot of various crops including alfalfa in China. Carbendazim was the most widely used fungicide in China to control alfalfa root rot (Liu et al., 2016). A study of 20 years ago showed that carbendazim reduced the *Fusarium* spp. (Min et al., 2002). However, in a recent study, *Fusarium* spp. were found to be resistant or intermediately sensitive to carbendazim, suggesting that carbendazim has failed to protect against root rot and resistance against carbendazim has emerged (Jiang et al., 2021). Recently in China mixture of fungicides, each having different modes of action, are being used to control root rot of many crops, including alfalfa. For example, seed treatment with the mixture of fludioxonil (phenylpyroles group) and difenoconazole (trizol group) in the 1:4 ratio demonstrated the best control efficiency at seedling and adult stages against root rot caused by *B. sorokiniana* (Wei et al., 2020). Fludioxonil inhibits glucose phosphorylation, while difenoconazole inhibits the C14-demethylase enzyme that participates in the ergosterol production of root rot pathogens (Wei et al., 2020). In the United States, metalaxyl and mefenoxam were widely used as a seed treatment or soil application to combat alfalfa root rot. These fungicides specifically target the ribosomal RNA polymerases of pathogens. However, resistance in oomycetes to these fungicides had emerged (Hwang et al., 1993). Quinone outside inhibitor (QoI) fungicides such as azoxystrobin and pyraclostrobin have also been widely used to manage alfalfa disease caused by root rot pathogens worldwide. These fungicides block the electron transport at the mitochondrial cytochrome oxidase bc1 complex, affecting respiration (Venancio et al., 2003). However, resistance to these fungicides has emerged in some pathogens due to a mutation at a target binding site (Bartlett et al., 2002). However, combined with other fungicides, these are still effective against root rot. For example, fungicides azoxystrobin and tebuconazole reduced 50 to 90% root rot caused by *R. solani* over three years when applied as a soil drench at 76 to 304 g a.i./ha and 250 g a.i./ha, respectively. In other countries, fungicides are combined with BCAs to control root rot. For example, a combination of azoxystrobin applied at 76 g a.i./ha and the *Bacillus* isolate MSU-127 reduced the crown and root rot disease and increase the yield remarkably (Kiewnick et al., 2001).

Besides, numerous phytochemicals, for example, steroids, tannins, flavonoids and alkaloids, have demonstrated antimicrobial activities against root rot (Lee et al., 2001). A product Biotos from *Gaultheria* spp. not only controlled root rot disease but also increase the yield. In another study, different concentrations of aqueous *Chenopodium album* extracts have been used to control root rot disease caused by *F. solani*. About 6% *C. album* extract reduced *Fusarium* root rot incidence from 47.49 to 28.25% (Abu-Tahon et al., 2021). The medicinal plants, i.e., *Prosopis africana*, *Anacardium occidentale* and *Nigella sativa* leaf and/whole plant parts extracts, have been assayed against root rot disease caused by *M phaseolina*, observing inhibition of its growth. Various alkaloids, saponins, tannins, flavonoids, anthraquinones, octadecadienoic acid, pentadecanoic acid, 1,2,3,4, butaneteterol, octadecanoic acid and linoleic acid were found in these extracts. In addition, *Lippia gracilis* oil extracts were found to suppress root rot disease.

Elicitors are natural or synthetic compounds, which induce systemic acquired resistance (SAR) and protect alfalfa from bacterial, fungal, and viral pathogens. The elicitors such as benzothiadiazole (BTH), chitosan (CHT), phenylalanine (PHE), and salicylic acid (SA), have been applied to control root rot in other plants (Pawlowski et al., 2016). The use of elicitors to stimulate the defensive system of alfalfa against root rot is currently unknown in China. In conclusion, since carbendazim and many fungicides are not species-specific, therefore it is likely that treated seeds though may protect the seeds from root rot pathogens but also may eliminate the keystone fungal species (Zotti et al., 2020). Fungicides applications as seed treatments can also affect endophytes which promote plant growth and protect plants from root rot pathogens. Besides, seed treatment could lead to loss of seed germination and reduction in early seedling development. If applied in high concentration can affect the plant metabolism. Usually, seeds are coated with fungicides and stored for long periods that results phytotoxicity (Lamichhane et al., 2020). The other main issue is that in many studies showed that fungicides significantly reduced the nodule formation in legume crops and affect their symbiotic association with mycorrhizal fungi (Mårtensson, 1992). In contrast to seed treatment, soil application is not detrimental to endophytes or root pathogens however it can disrupt the carbon and nitrogen cycling in soil, soil respiration and affect not target organisms. Furthermore, soil or seed application with one fungicide may be effective against single pathogen involve in root rot complex but not against diversity of pathogens. Combination of two or more fungicides having different mode of action could reduce the root rot effectively though it would be unbearable to farmers to bear costs of fungicides. The best strategy would be the combination of fungicide with BCAs because not only the amount of fungicide will be lowered, but pollution can also be reduced. Besides, both BCAs and fungicides both reduce the risk of the occurrence of fungicide resistance and improve the reliability of disease control compared with that provided using a biocontrol agent/fungicide alone.

## Conclusion, issues, and future perspectives

In conclusion, due to the involvement of diverse pathogens, the management of alfalfa root rot is exceptionally challenging. *Fusarium* spp. and *R. solani* are playing a dominant role involving other pathogens in causing root rot in different regions of China. However, large-scale isolation, systematic identification, and pathogenicity evaluation of the pathogens causing alfalfa root rot are still lacking in all major alfalfa-growing regions of China. Several alfalfa breeding lines or cultivars that show partial resistance to root rot have been screened. On the other hand, resistant responses are quantitative and governed by several genes. It is challenging to develop a cultivar that could resist diverse pathogens. Furthermore, alfalfa is a cross-pollinated autotetraploid with different breeding and selection requirements than diploid plants and pathogens with genetic complexities. Also, the disease severity resulting from root rot pathogens varies with location and years due to alterations in climatic (moisture and temperature), soil factors (compaction), host and pathogens factors. Besides, it is tough to assess the yield losses because roots are rarely examined unless foliar symptoms appear. In addition, most of the studies on the root rot pathogens are conducted in controlled environmental conditions. The capacity of a pathogen to cause root rot in alfalfa under such controlled conditions is not directly related to what happens to alfalfa in the pastures. Hence information of the controlled conditions environments experiments cannot be reliably correlated to pastures. Besides fungal and oomycetes pathogens, there is also a need to assess other soil-borne pathogens such as nematodes. All these data obtained will be used to control the root rot rationally. Moreover, investigations on alfalfa resistance QTLs are limited, and the genes that cause alfalfa resistance are unknown. There is a need to focus on research into alfalfa’s disease resistance mechanism against various pathogenic strains of the pathogens and look for the broad-spectrum and specificity of alfalfa against these pathogenic strains. In addition, identification of resistance genes to breed alfalfa resistant varieties to root rot and promote the sustainable production of alfalfa is also required. Furthermore, because most studies regarding alfalfa disease resistance use a single strain, it’s impossible to tell whether the cultivars chosen are disease-resistant in general. To comprehensively investigate disease resistance and screen a broad spectrum of varieties, there is a need to standardize disease resistance evaluation criteria for different pathogenic species and understand alfalfa resistance mechanisms to the strains within and between pathogens species. Furthermore, alfalfa’s pathogenic mechanisms, particularly its molecular basis, is still unknown. Future research should include genome sequencing and comparative analysis of different strains within and within species of the pathogen that causes alfalfa root rot and transcriptomics investigations of the genes expressed by different strains during the infection of alfalfa. Currently, farmers rely solely on fungicides as seed treatments and/or soil sprays, but pathogen populations have developed resistance to fungicides. Biological control agents, such as arbuscular fungi, *Bacillus* and *Trichoderma* spp. have recently been utilized to prevent alfalfa root rot. On the other hand, these biological control agents present challenges in terms of field survival, proliferation, growth, and adaptability. Furthermore, each pathogen that causes root rot produces distinct spores, all of which contribute to the disease’s occurrence and spread. The number of spores and their ability to survive directly impact the disease’s prevalence and severity. The ideal climatic conditions for the formation and germination of many types of spores, on the other hand, are unknown. Simultaneously, studying spore production and germination mechanisms and their major regulatory factors is required to offer a theoretical foundation for developing innovative alfalfa root rot prevention and control approaches. In the future, high-throughput phenotyping and genotyping technologies, genomic methods, genome-wide investigations, transcriptomics, and next-generation sequencing techniques will make it possible to find root rot resistant cultivars and better understand root rot pathogen pathogenesis. These techniques will also aid in the discovery of genes linked to alfalfa resistance or susceptibility to root rot pathogens.

## Author contributions

AA and MM: writing original draft and software and figure preparations. MAS, MKS, BH, SN, and BK: collecting literatures and tables preparations and editing. XF: validation, formal analysis, and finalized the review. LZ: supervision, project administration, resources, and funding acquisition. All authors contributed to the article and approved the submitted version.

## Funding

This work was supported by the High-talent Introduction and Continuous Training Fund to LZ (grant no: 10300000021LL05) and Discipline Construction Funds (grant no: 10407000019CC2213G), supported by Zhejiang Academy of Agricultural Sciences (ZAAS) and State Key Laboratory for Managing Biotic and Chemical Threats to the Quality and Safety of Agro-products (10417000022CE0601G/029).

## Conflict of interest

The authors declare that the research was conducted in the absence of any commercial or financial relationships that could be construed as a potential conflict of interest.

## Publisher’s note

All claims expressed in this article are solely those of the authors and do not necessarily represent those of their affiliated organizations, or those of the publisher, the editors and the reviewers. Any product that may be evaluated in this article, or claim that may be made by its manufacturer, is not guaranteed or endorsed by the publisher.

## Footnotes

^1^ www.medicagohapmap.org/
